# Supplementary material for: TopBP1 utilises a bipartite GINS binding mode to support genome replication
Source: Nat Commun. 2024 Feb 27;15:1797. doi: 10.1038/s41467-024-45946-0 (PMC10899662; doi:10.1038/s41467-024-45946-0)
Supplement: Supplementary file 3 — Description of Additional Supplementary Files [file 41467_2024_45946_MOESM3_ESM.pdf]

Description of Additional Supplementary Files

File Name: Supplementary Data 1
Description: **List of crosslinked GINI region peptides found by mass spectrometry** (experiment supplementary Fig.S10).

File Name: Supplementary Data 2
Description: **List with statistical analysis of peptides found on chromatin by CHROMASS** (experiment Fig. 6a-c, Supplementary Fig. 14).

File Name: Supplementary Data 3
Description: **Detailed information of antibodies (sheet 1) and plasmids (sheet 2) used in this study.**
