# Supplementary material for: TopBP1 utilises a bipartite GINS binding mode to support genome replication
Source: Nat Commun. 2024 Feb 27;15:1797. doi: 10.1038/s41467-024-45946-0 (PMC10899662; doi:10.1038/s41467-024-45946-0)
Supplement: Supplementary file 4 — Supplementary Data 1 [file 41467_2024_45946_MOESM4_ESM.pdf]

Number of PSM's for

Cross link **IGD**	Search engine	Scan Number	#PSM's total
TopBP11-766-strep (482) PsfI (61)	pLink2	BT06_15829	1
TopBP11-766-strep (480) PsfI (63)	MM	BT06_15955_15955	2
TopBP11-766-strep (475) PsfI (63)	MM	BT05_13950_13939_13952<br>BT06_14182<br>BT07_13724	3<br>1<br>1
	pLink2	BT04_13388<br>BT05_13939_13952<br>BT06_14182<br>BT07_13713	1<br>2<br>1<br>1
TopBP11-766-strep (468) PsfI (63)	pLink2	BT05_12836<br>BT06_12998	1<br>1
TopBP11-766-strep (466) PsfI (63)	pLink2	BT05_14419<br>BT06_14715<br>BT07_14217	1<br>1<br>1
TopBP11-766-strep (475) PsfIII (80)	pLink2	BT06_10244	1
TopBP11-766-strep (474) PsfIII (80)	pLink2	BT06_9156	1
IGD TopBP11-766-strep (473) PsfIII (80)	pLink2	BT05_9151	1
TopBP11-766-strep (468) PsfIII (80)	pLink2	BT05_8482<br>BT06_8457	1<br>1
TopBP11-766-strep (466) PsfIII (80)	pLink2	BT05_10736<br>BT07_10624	1<br>1

Cross link **ISD**	Search engine	Scan Number	#PSM's total
TopBP11-766-strep (482) PsfI (61)	MM	BT01_45717	1
TopBP11-766-strep (480) PsfI (63)	MM	BT01_45733	1
TopBP11-766-strep (475) PsfI (63)	pLink2	BT01_33941_33922	2
TopBP11-766-strep (468) PsfI (63)	pLink2	BT01_29586_29534_29519	3
TopBP11-766-strep (466) PsfI (63)	pLink2	BT01_35956	1
TopBP11-766-strep (480) PsfIII (80)	pLink2	BT01_22561	1
TopBP11-766-strep (475) PsfIII (80)	pLink2	BT01_21105_21106	2
TopBP11-766-strep (474) PsfIII (80)	pLink2	BT01_17827	1
TopBP11-766-strep (468) PsfIII (80)	pLink2	BT01_16312_16316	2
TopBP11-766-strep (466) PsfIII (80)	pLink2	BT01_21980	1
TopBP11-766-strep (480) PsfIII (74)	MM	BT01_57130	1

ISD= in-solution digest; IGD= in-gel digest

IGD TopBP11-766-strep (482) PsfI (61)

DFAPSEKHEQADEDLLSQYENGSSTVVEAK
|
SDLIPTIKFR

DFAPSEKHEQADEDLLSQYENGSSTVVEAK, MH+ 4650.2734, m/z 930.8605

File: ACE_0704_BT06-calib.mzML, Scan: 15829, Exp. m/z: 931.4592095799338, Charge: 5

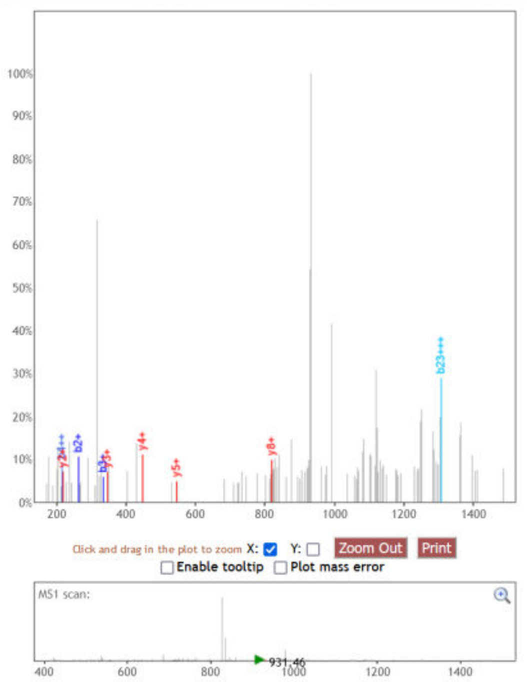

b+	b2+	b3+	#	Seq	#	y+	y2+	y3+
116.0342	58.5207	39.3496	1	D	30			
263.1026	132.0550	88.3724	2	F	29	4535.2464	2268.1268	1512.4203
334.1397	167.5735	112.0514	3	A	28	4388.1780	2194.5926	1463.3975
431.1925	216.0999	144.4024	4	P	27	4317.1409	2159.0741	1439.7185
518.2245	259.6159	173.4130	5	S	26	4220.0881	2110.5477	1407.3676
647.2671	324.1372	216.4272	6	E	25	4133.0561	2067.0317	1378.3569
2102.1168	1051.5621	701.3771	7	K	24	4004.0135	2002.5104	1335.3427
2239.1757	1120.0915	747.0634	8	H	23	2549.1638	1275.0855	850.3928
2368.2183	1184.6128	790.0776	9	E	22	2412.1049	1206.5561	804.7065
2496.2769	1248.6421	832.7638	10	Q	21	2283.0623	1142.0348	761.6923
2567.3140	1284.1607	856.4429	11	A	20	2155.0037	1078.0055	719.0061
2682.3410	1341.6741	894.7852	12	D	19	2083.9666	1042.4869	695.3271
2811.3836	1406.1954	937.7994	13	E	18	1968.9397	984.9735	656.9847
2926.4105	1463.7089	976.1417	14	D	17	1839.8971	920.4522	613.9705
3039.4946	1520.2509	1013.8364	15	L	16	1724.8701	862.9387	575.6282
3152.5786	1576.7930	1051.5311	16	L	15	1611.7861	806.3967	537.9335
3239.6107	1620.3090	1080.5417	17	S	14	1498.7020	749.8546	500.2388
3367.6692	1684.3383	1123.2279	18	Q	13	1411.6700	706.3386	471.2282
3530.7326	1765.8699	1177.5824	19	Y	12	1283.6114	642.3093	428.5420
3659.7752	1830.3912	1220.5966	20	E	11	1120.5481	560.7777	374.1875
3773.8181	1887.4127	1258.6109	21	N	10	991.5055	496.2564	331.1733
3830.8396	1915.9234	1277.6180	22	G	9	877.4625	439.2349	293.1590
3917.8716	1959.4394	1306.6287	23	S	8	820.4411	410.7242	274.1519
4004.9036	2002.9554	1335.6394	24	S	7	733.4090	367.2082	245.1412
4105.9513	2053.4793	1369.3219	25	T	6	646.3770	323.6921	216.1305
4205.0197	2103.0135	1402.3448	26	V	5	545.3293	273.1683	182.4480
4304.0881	2152.5477	1435.3676	27	V	4	446.2609	223.6341	149.4252
4433.1307	2217.0690	1478.3818	28	E	3	347.1925	174.0999	116.4024
4504.1678	2252.5876	1502.0608	29	A	2	218.1499	109.5786	73.3882
			30	K	1	147.1128	74.0600	49.7091

[Click] to move table

Linked Peptide + Linker Mass: 1326.75
Linker Mass: 138.06808

SDLIPTIKFR, MH+ 4650.2734, m/z 930.8605

File: ACE_0704_BT06-calib.mzML, Scan: 15829, Exp. m/z: 931.4592095799338, Charge: 5

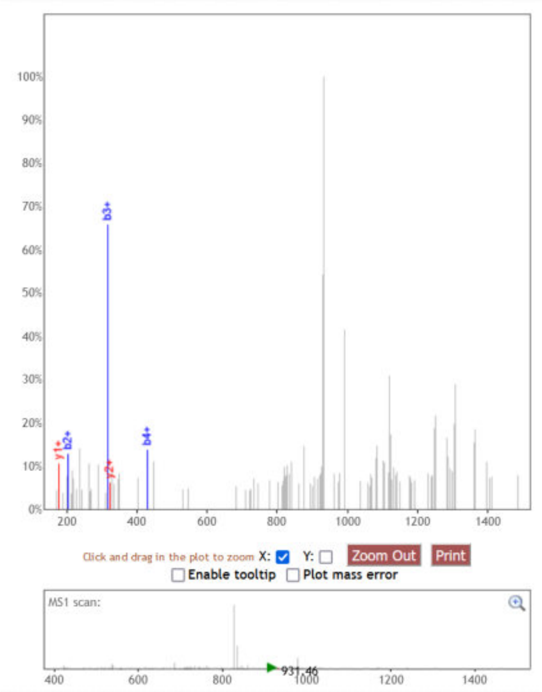

b+	b2+	b3+	#	Seq	#	y+	y2+	y3+
88.0393	44.5233	30.0180	1	S	10			
203.0662	102.0368	68.3603	2	D	9	4563.2413	2282.1243	1521.7520
316.1503	158.5788	106.0550	3	L	8	4448.2144	2224.6108	1483.4096
429.2344	215.1208	143.7496	4	I	7	4335.1303	2168.0688	1445.7150
526.2871	263.6472	176.1006	5	P	6	4222.0463	2111.5268	1408.0203
4087.9142	2044.4608	1363.3096	6	T	5	4124.9935	2063.0004	1375.6693
4200.9983	2101.0028	1401.0043	7	I	4	563.3664	282.1868	188.4603
4329.0933	2165.0503	1443.7026	8	K	3	450.2823	225.6448	150.7656
4476.1617	2238.5845	1492.7254	9	F	2	322.1874	161.5973	108.0673
			10	R	1	175.1190	88.0631	59.0445

[Click] to move table

Linked Peptide + Linker Mass: 3460.58
Linker Mass: 138.06808

IGD   TopBP11-766-strep (480)   PsfI (63)

```
DFAPSEKHEQADEDLLSQYENGSSTVVEAK
     |
SDLIPTIKFR
```

DFAPSEKHEQADEDLLSQYENGSSTVVEAK, MH+ 4650.2734, m/z 930.8605

File: ACE_0704_BT06-calib.mzML, Scan: 15955, Exp. m/z: 931.2626829743398, Charge: 5

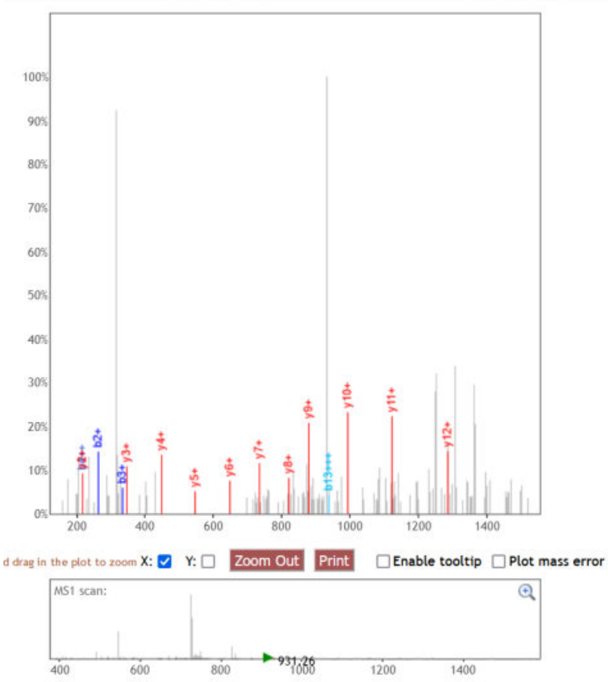

b+	b2+	b3+	#	Seq	#	y+	y2+	y3+
116.0342	58.5207	39.3496	1	D	30			
263.1026	132.0550	88.3724	2	F	29	4535.2464	2268.1268	1512.4203
334.1397	167.5735	112.0514	3	A	28	4388.1780	2194.5926	1463.3975
431.1925	216.0999	144.4024	4	P	27	4317.1409	2159.0741	1439.7185
1844.9793	922.9933	615.6646	5	S	26	4220.0881	2110.5477	1407.3676
1974.0219	987.5146	658.6788	6	E	25	2806.3014	1403.6543	936.1053
2102.1168	1051.5621	701.3771	7	K	24	2677.2588	1339.1330	893.0911
2239.1757	1120.0915	747.0634	8	H	23	2549.1638	1275.0855	850.3928
2368.2183	1184.6128	790.0776	9	E	22	2412.1049	1206.5561	804.7065
2496.2769	1248.6421	832.7638	10	Q	21	2283.0623	1142.0348	761.6923
2567.3140	1284.1607	856.4429	11	A	20	2155.0037	1078.0055	719.0061
2682.3410	1341.6741	894.7852	12	D	19	2083.9666	1042.4869	695.3271
2811.3836	1406.1954	937.7994	13	E	18	1968.9397	984.9735	656.9847
2926.4105	1463.7089	976.1417	14	D	17	1839.8971	920.4522	613.9705
3039.4946	1520.2509	1013.8364	15	L	16	1724.8701	862.9387	575.6282
3152.5786	1576.7930	1051.5311	16	L	15	1611.7861	806.3967	537.9335
3239.6107	1620.3090	1080.5417	17	S	14	1498.7020	749.8546	500.2388
3367.6692	1684.3383	1123.2279	18	Q	13	1411.6700	706.3386	471.2282
3530.7326	1765.8699	1177.5824	19	Y	12	1283.6114	642.3093	428.5420
3659.7752	1830.3912	1220.5966	20	E	11	1120.5481	560.7777	374.1875
3773.8181	1887.4127	1258.6109	21	N	10	991.5055	496.2564	331.1733
3830.8396	1915.9234	1277.6180	22	G	9	877.4625	439.2349	293.1590
3917.8716	1959.4394	1306.6287	23	S	8	820.4411	410.7242	274.1519
4004.9036	2002.9554	1335.6394	24	S	7	733.4090	367.2082	245.1412
4105.9513	2053.4793	1369.3219	25	T	6	646.3770	323.6921	216.1305
4205.0197	2103.0135	1402.3448	26	V	5	545.3293	273.1683	182.4480
4304.0881	2152.5477	1435.3676	27	V	4	446.2609	223.6341	149.4252
4433.1307	2217.0690	1478.3818	28	E	3	347.1925	174.0999	116.4024
4504.1678	2252.5876	1502.0608	29	A	2	218.1499	109.5786	73.3882
			30	K	1	147.1128	74.0600	49.7091

[Click] to move table

Linked Peptide + Linker Mass: 1326.75
Linker Mass: 138.06808

SDLIPTIKFR, MH+ 4650.2734, m/z 930.8605

File: ACE_0704_BT06-calib.mzML, Scan: 15955, Exp. m/z: 931.2626829743398, Charge: 5

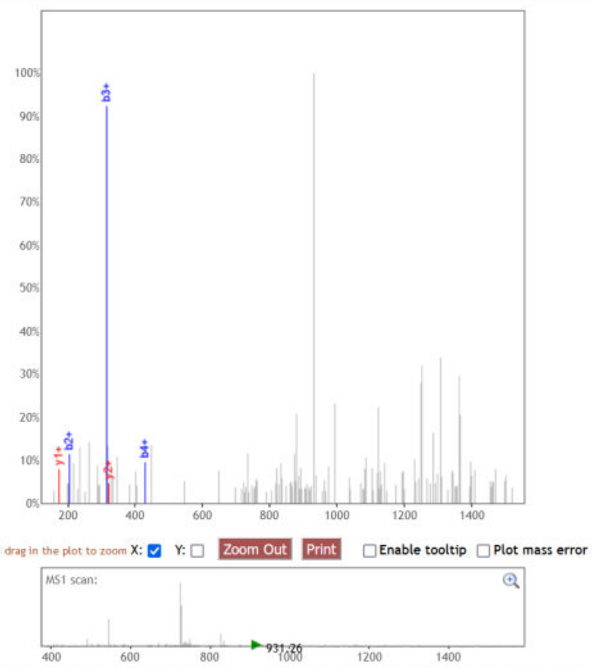

b+	b2+	b3+	#	Seq	#	y+	y2+	y3+
88.0393	44.5233	30.0180	1	S	10			
203.0662	102.0368	68.3603	2	D	9	4563.2413	2282.1243	1521.7520
316.1503	158.5788	106.0550	3	L	8	4448.2144	2224.6108	1483.4096
429.2344	215.1208	143.7496	4	I	7	4335.1303	2168.0688	1445.7150
526.2871	263.6472	176.1006	5	P	6	4222.0463	2111.5268	1408.0203
627.3348	314.1710	209.7831	6	T	5	4124.9935	2063.0004	1375.6693
740.4189	370.7131	247.4778	7	I	4	4023.9458	2012.4765	1341.9868
4329.0933	2165.0503	1443.7026	8	K	3	3910.8617	1955.9345	1304.2921
4476.1617	2238.5845	1492.7254	9	F	2	322.1874	161.5973	108.0673
			10	R	1	175.1190	88.0631	59.0445

[Click] to move table

Linked Peptide + Linker Mass: 3460.58
Linker Mass: 138.06808

IGD TopBP11-766-strep (475) PsfI (63)

```
    KDFAPSEK
    |
SDLIPTIKFR
```

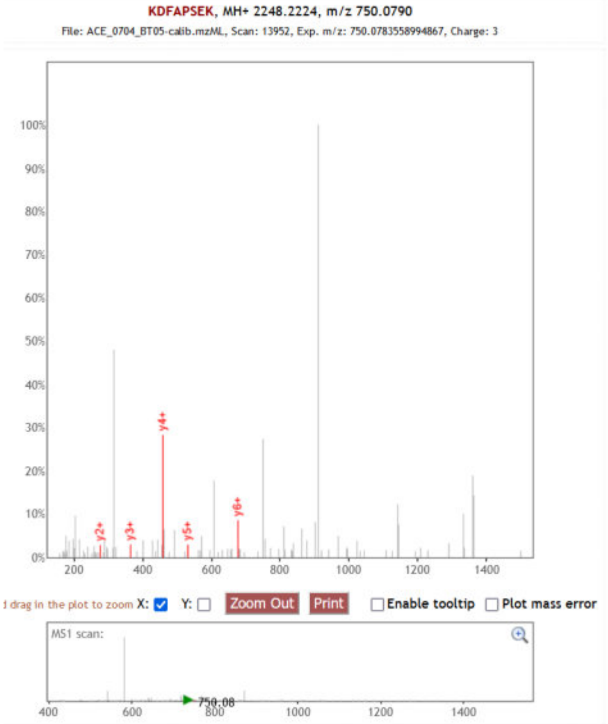

b+	b2+	#	Seq	#	y+	y2+
1455.8570	728.4321	1	K	8		
1570.8839	785.9456	2	D	7	793.3727	397.1900
1717.9523	859.4798	3	F	6	678.3457	339.6765
1788.9894	894.9984	4	A	5	531.2773	266.1423
1886.0422	943.5247	5	P	4	460.2402	230.6237
1973.0742	987.0408	6	S	3	363.1874	182.0974
2102.1168	1051.5621	7	E	2	276.1554	138.5813
		8	K	1	147.1128	74.0600

[Click] to move table

Linked Peptide + Linker Mass: 1326.75
Linker Mass: 138.06808

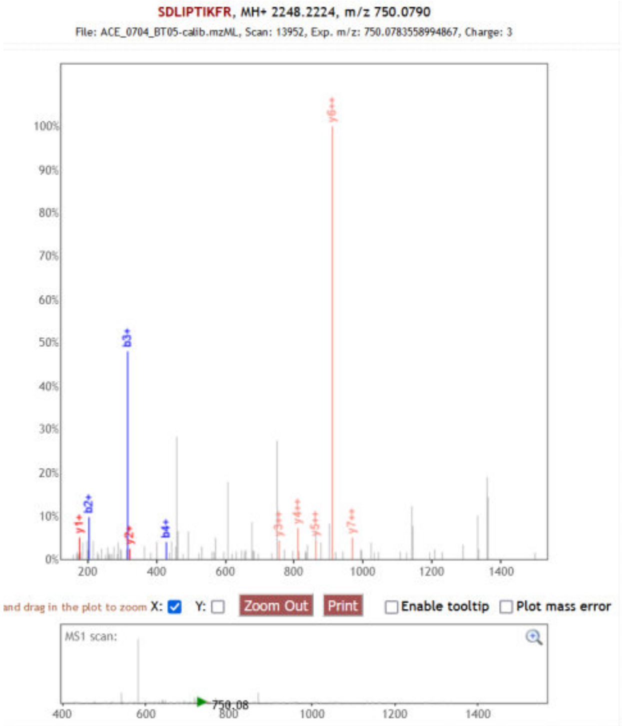

b+	b2+	#	Seq	#	y+	y2+
88.0393	44.5233	1	S	10		
203.0662	102.0368	2	D	9	2161.1903	1081.0988
316.1503	158.5788	3	L	8	2046.1634	1023.5853
429.2344	215.1208	4	I	7	1933.0793	967.0433
526.2871	263.6472	5	P	6	1819.9953	910.5013
627.3348	314.1710	6	T	5	1722.9425	861.9749
740.4189	370.7131	7	I	4	1621.8948	811.4510
1927.0423	964.0248	8	K	3	1508.8108	754.9090
2074.1107	1037.5590	9	F	2	322.1874	161.5973
		10	R	1	175.1190	88.0631

[Click] to move table

Linked Peptide + Linker Mass: 1058.53
Linker Mass: 138.06808

IGD TopBP11-766-strep (468) PsfI (63)

```
     KNSSFSK
      |
SDLIPTIKFR
```

KNSSFSK, MH+ 2124.1699, m/z 708.7281

File: ACE_0704_BT06.mzML, Scan: 12998, Exp. m/z: 708.728759765625, Charge: 3

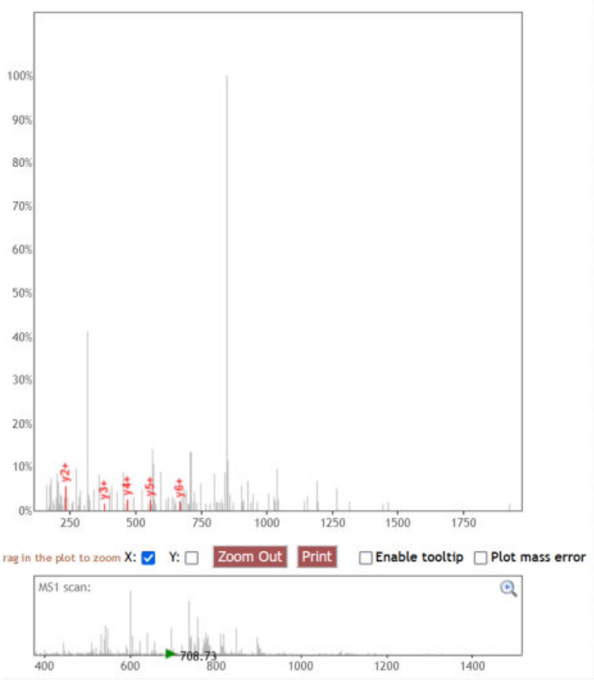

b+	b2+	#	Seq	#	y+	y2+
1455.8569	728.4321	1	K	7		
1569.8998	785.4536	2	N	6	669.3202	335.1638
1656.9319	828.9696	3	S	5	555.2773	278.1423
1743.9639	872.4856	4	S	4	468.2453	234.6263
1891.0323	946.0198	5	F	3	381.2132	191.1103
1978.0643	989.5358	6	S	2	234.1448	117.5761
		7	K	1	147.1128	74.0600

[Click] to move table

Static Modifications:
C: 57.0215
Linked Peptide + Linker Mass: 1326.75
Linker Mass: 138.068

SDLIPTIKFR, MH+ 2124.1699, m/z 708.7281

File: ACE_0704_BT06.mzML, Scan: 12998, Exp. m/z: 708.728759765625, Charge: 3

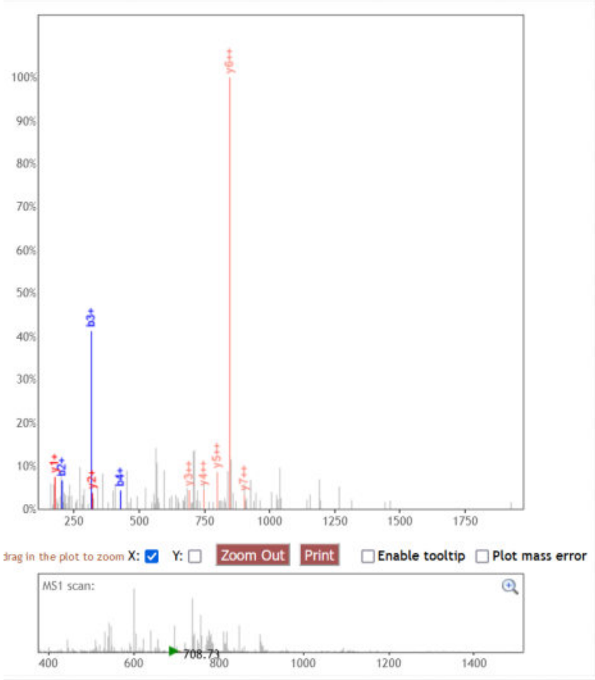

b+	b2+	#	Seq	#	y+	y2+
88.0393	44.5233	1	S	10		
203.0662	102.0368	2	D	9	2037.1378	1019.0725
316.1503	158.5788	3	L	8	1922.1109	961.5591
429.2344	215.1208	4	I	7	1809.0268	905.0170
526.2871	263.6472	5	P	6	1695.9428	848.4750
627.3348	314.1710	6	T	5	1598.8900	799.9486
740.4189	370.7131	7	I	4	1497.8423	749.4248
1802.9898	901.9985	8	K	3	1384.7582	692.8828
1950.0582	975.5327	9	F	2	322.1874	161.5973
		10	R	1	175.1190	88.0631

[Click] to move table

Static Modifications:
C: 57.0215
Linked Peptide + Linker Mass: 934.48
Linker Mass: 138.068

IGD   TopBP11-766-strep (466)   PsfI (63)

```
    AALLKK
         |
SDLIPTIKFR
```

AALLKK, MH+ 1970.2048, m/z 657.4064

File: ACE_0704_BT06.mzML, Scan: 14715, Exp. m/z: 657.406433105469, Charge: 3

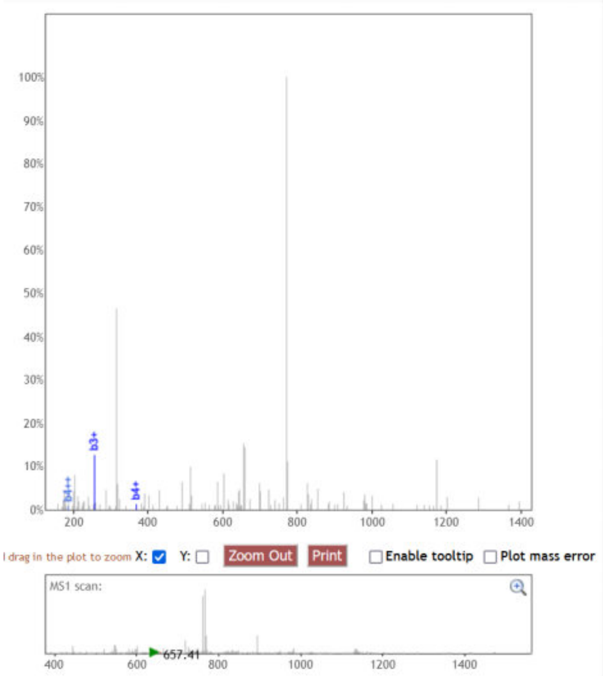

b+	b2+	#	Seq	#	y+	y2+
72.0444	36.5258	1	A	6		
143.0815	72.0444	2	A	5	1899.1677	950.0875
256.1656	128.5864	3	L	4	1828.1306	914.5689
369.2496	185.1285	4	L	3	1715.0465	858.0269
1824.0993	912.5533	5	K	2	1601.9624	801.4849
		6	K	1	147.1128	74.0600

[Click] to move table

Static Modifications:
C: 57.0215
Linked Peptide + Linker Mass: 1326.75
Linker Mass: 138.068

SDLIPTIKFR, MH+ 1970.2048, m/z 657.4064

File: ACE_0704_BT06.mzML, Scan: 14715, Exp. m/z: 657.406433105469, Charge: 3

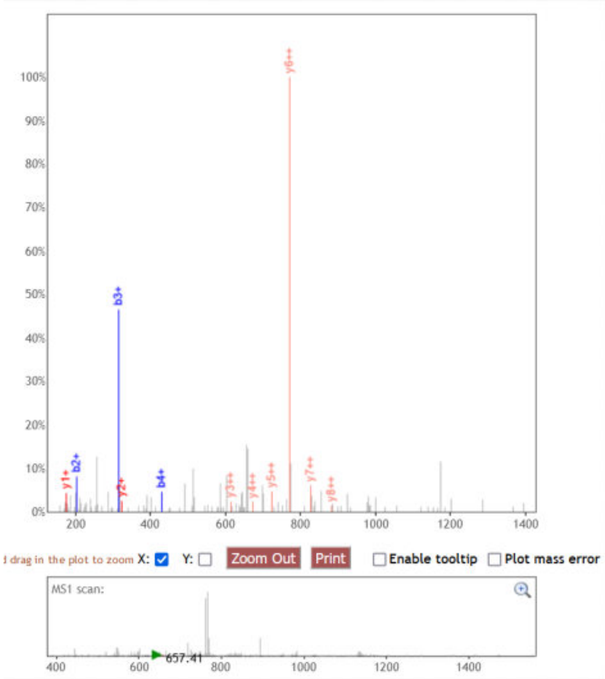

b+	b2+	#	Seq	#	y+	y2+
88.0393	44.5233	1	S	10		
203.0662	102.0368	2	D	9	1883.1728	942.0900
316.1503	158.5788	3	L	8	1768.1458	884.5765
429.2344	215.1208	4	I	7	1655.0617	828.0345
526.2871	263.6472	5	P	6	1541.9777	771.4925
627.3348	314.1710	6	T	5	1444.9249	722.9661
740.4189	370.7131	7	I	4	1343.8772	672.4423
1649.0247	825.0160	8	K	3	1230.7932	615.9002
1796.0931	898.5502	9	F	2	322.1874	161.5973
		10	R	1	175.1190	88.0631

[Click] to move table

Static Modifications:
C: 57.0215
Linked Peptide + Linker Mass: 780.51
Linker Mass: 138.068

ISD TopBP11-766-strep (482) PsfI (61)

DFAPSEKHEQADEDLLSQYENGSSTVVEAK
|
SDLIPTIKFR

DFAPSEKHEQADEDLLSQYENGSSTVVEAK, MH+ 4650.2734, m/z 1163.3238

File: ACE_0704_BT01_StageTip_210min-calib.mzML, Scan: 45717, Exp. m/z: 1163.5731668656674, Charge: 4

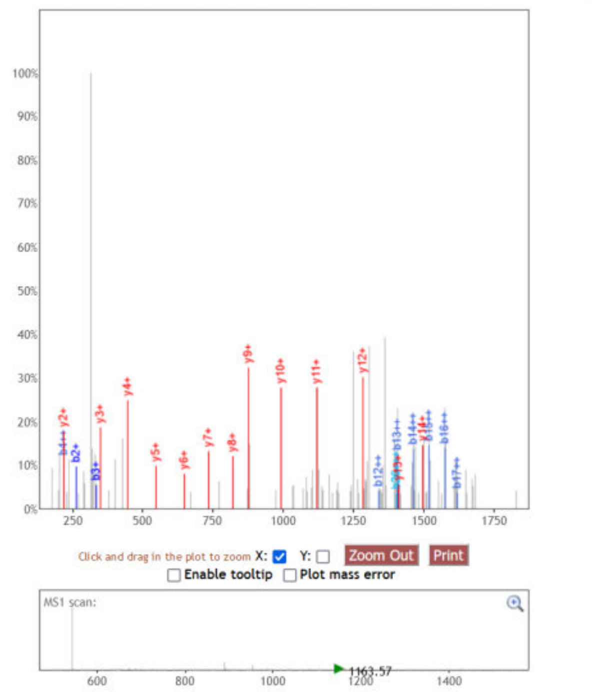

b+	b2+	b3+	#	Seq	#	y+	y2+	y3+
116.0342	58.5207	39.3496	1	D	30			
263.1026	132.0550	88.3724	2	F	29	4535.2464	2268.1268	1512.4203
334.1397	167.5735	112.0514	3	A	28	4388.1780	2194.5926	1463.3975
431.1925	216.0999	144.4024	4	P	27	4317.1409	2159.0741	1439.7185
518.2245	259.6159	173.4130	5	S	26	4220.0881	2110.5477	1407.3676
647.2671	324.1372	216.4272	6	E	25	4133.0561	2067.0317	1378.3569
2102.1168	1051.5621	701.3771	7	K	24	4004.0135	2002.5104	1335.3427
2239.1757	1120.0915	747.0634	8	H	23	2549.1638	1275.0855	850.3928
2368.2183	1184.6128	790.0776	9	E	22	2412.1049	1206.5561	804.7065
2496.2769	1248.6421	832.7638	10	Q	21	2283.0623	1142.0348	761.6923
2567.3140	1284.1607	856.4429	11	A	20	2155.0037	1078.0055	719.0061
2682.3410	1341.6741	894.7852	12	D	19	2083.9666	1042.4869	695.3271
2811.3836	1406.1954	937.7994	13	E	18	1968.9397	984.9735	656.9847
2926.4105	1463.7089	976.1417	14	D	17	1839.8971	920.4522	613.9705
3039.4946	1520.2509	1013.8364	15	L	16	1724.8701	862.9387	575.6282
3152.5786	1576.7930	1051.5311	16	L	15	1611.7861	806.3967	537.9335
3239.6107	1620.3090	1080.5417	17	S	14	1498.7020	749.8546	500.2388
3367.6692	1684.3383	1123.2279	18	Q	13	1411.6700	706.3386	471.2282
3530.7326	1765.8699	1177.5824	19	Y	12	1283.6114	642.3093	428.5420
3659.7752	1830.3912	1220.5966	20	E	11	1120.5481	560.7777	374.1875
3773.8181	1887.4127	1258.6109	21	N	10	991.5055	496.2564	331.1733
3830.8396	1915.9234	1277.6180	22	G	9	877.4625	439.2349	293.1590
3917.8716	1959.4394	1306.6287	23	S	8	820.4411	410.7242	274.1519
4004.9036	2002.9554	1335.6394	24	S	7	733.4090	367.2082	245.1412
4105.9513	2053.4793	1369.3219	25	T	6	646.3770	323.6921	216.1305
4205.0197	2103.0135	1402.3448	26	V	5	545.3293	273.1683	182.4480
4304.0881	2152.5477	1435.3676	27	V	4	446.2609	223.6341	149.4252
4433.1307	2217.0690	1478.3818	28	E	3	347.1925	174.0999	116.4024
4504.1678	2252.5876	1502.0608	29	A	2	218.1499	109.5786	73.3882
			30	K	1	147.1128	74.0600	49.7091

[Click] to move table

Linked Peptide + Linker Mass: 1326.75
Linker Mass: 138.06808

SDLIPTIKFR, MH+ 4650.2734, m/z 1163.3238

File: ACE_0704_BT01_StageTip_210min-calib.mzML, Scan: 45717, Exp. m/z: 1163.5731668656674, Charge: 4

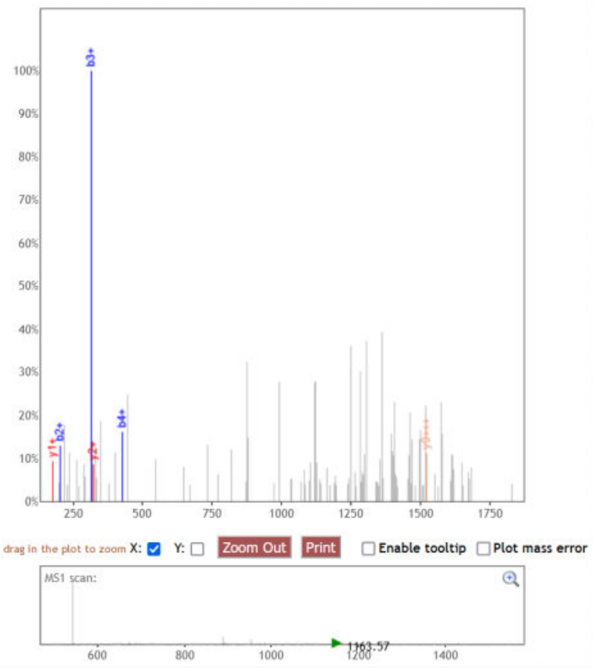

b+	b2+	b3+	#	Seq	#	y+	y2+	y3+
88.0393	44.5233	30.0180	1	S	10			
203.0662	102.0368	68.3603	2	D	9	4563.2413	2282.1243	1521.7520
316.1503	158.5788	106.0550	3	L	8	4448.2144	2224.6108	1483.4096
429.2344	215.1208	143.7496	4	I	7	4335.1303	2168.0688	1445.7150
526.2871	263.6472	176.1006	5	P	6	4222.0463	2111.5268	1408.0203
4087.9142	2044.4608	1363.3096	6	T	5	4124.9935	2063.0004	1375.6693
4200.9983	2101.0028	1401.0043	7	I	4	563.3664	282.1868	188.4603
4329.0933	2165.0503	1443.7026	8	K	3	450.2823	225.6448	150.7656
4476.1617	2238.5845	1492.7254	9	F	2	322.1874	161.5973	108.0673
			10	R	1	175.1190	88.0631	59.0445

[Click] to move table

Linked Peptide + Linker Mass: 3460.58
Linker Mass: 138.06808

ISD TopBP11-766-strep (480) PsfI (63)

DFAPSEKHEQADEDLLSQYENGSSTVVEAK

|

SDLIPTIKFR

DFAPSEKHEQADEDLLSQYENGSSTVVEAK, MH+ 4650.2734, m/z 1163.3238

File: ACE_0704_BT01_StageTip_210min-calib.mzML, Scan: 45733, Exp. m/z: 1163.825097881888, Charge: 4

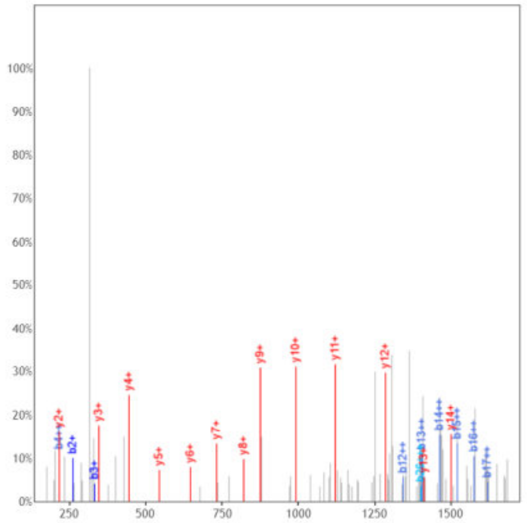

drag in the plot to zoom X: Y: Zoom Out Print Enable tooltip Plot mass error

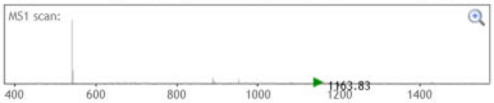

b+	b2+	b3+	#	Seq	#	y+	y2+	y3+
116.0342	58.5207	39.3496	1	D	30			
263.1026	132.0550	88.3724	2	F	29	4535.2464	2268.1268	1512.4203
334.1397	167.5735	112.0514	3	A	28	4388.1780	2194.5926	1463.3975
431.1925	216.0999	144.4024	4	P	27	4317.1409	2159.0741	1439.7185
1844.9793	922.9933	615.6646	5	S	26	4220.0881	2110.5477	1407.3676
1974.0219	987.5146	658.6788	6	E	25	2806.3014	1403.6543	936.1053
2102.1168	1051.5621	701.3771	7	K	24	2677.2588	1339.1330	893.0911
2239.1757	1120.0915	747.0634	8	H	23	2549.1638	1275.0855	850.3928
2368.2183	1184.6128	790.0776	9	E	22	2412.1049	1206.5561	804.7065
2496.2769	1248.6421	832.7638	10	Q	21	2283.0623	1142.0348	761.6923
2567.3140	1284.1607	856.4429	11	A	20	2155.0037	1078.0055	719.0061
2682.3410	1341.6741	894.7852	12	D	19	2083.9666	1042.4869	695.3271
2811.3836	1406.1954	937.7994	13	E	18	1968.9397	984.9735	656.9847
2926.4105	1463.7089	976.1417	14	D	17	1839.8971	920.4522	613.9705
3039.4946	1520.2509	1013.8364	15	L	16	1724.8701	862.9387	575.6282
3152.5786	1576.7930	1051.5311	16	L	15	1611.7861	806.3967	537.9335
3239.6107	1620.3090	1080.5417	17	S	14	1498.7020	749.8546	500.2388
3367.6692	1684.3383	1123.2279	18	Q	13	1411.6700	706.3386	471.2282
3530.7326	1765.8699	1177.5824	19	Y	12	1283.6114	642.3093	428.5420
3659.7752	1830.3912	1220.5966	20	E	11	1120.5481	560.7777	374.1875
3773.8181	1887.4127	1258.6109	21	N	10	991.5055	496.2564	331.1733
3830.8396	1915.9234	1277.6180	22	G	9	877.4625	439.2349	293.1590
3917.8716	1959.4394	1306.6287	23	S	8	820.4411	410.7242	274.1519
4004.9036	2002.9554	1335.6394	24	S	7	733.4090	367.2082	245.1412
4105.9513	2053.4793	1369.3219	25	T	6	646.3770	323.6921	216.1305
4205.0197	2103.0135	1402.3448	26	V	5	545.3293	273.1683	182.4480
4304.0881	2152.5477	1435.3676	27	V	4	446.2609	223.6341	149.4252
4433.1307	2217.0690	1478.3818	28	E	3	347.1925	174.0999	116.4024
4504.1678	2252.5876	1502.0608	29	A	2	218.1499	109.5786	73.3882
			30	K	1	147.1128	74.0600	49.7091

[Click] to move table

Linked Peptide + Linker Mass: 1326.75

Linker Mass: 138.06808

SDLIPTIKFR, MH+ 4650.2734, m/z 1163.3238

File: ACE_0704_BT01_StageTip_210min-calib.mzML, Scan: 45733, Exp. m/z: 1163.825097881888, Charge: 4

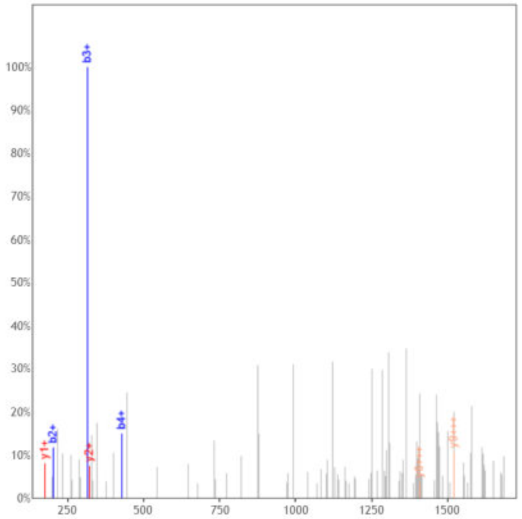

drag in the plot to zoom X: Y: Zoom Out Print Enable tooltip Plot mass error

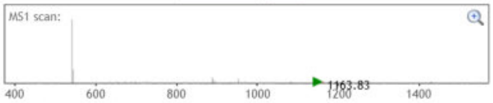

b+	b2+	b3+	#	Seq	#	y+	y2+	y3+
88.0393	44.5233	30.0180	1	S	10			
203.0662	102.0368	68.3603	2	D	9	4563.2413	2282.1243	1521.7520
316.1503	158.5788	106.0550	3	L	8	4448.2144	2224.6108	1483.4096
429.2344	215.1208	143.7496	4	I	7	4335.1303	2168.0688	1445.7150
526.2871	263.6472	176.1006	5	P	6	4222.0463	2111.5268	1408.0203
627.3348	314.1710	209.7831	6	T	5	4124.9935	2063.0004	1375.6693
740.4189	370.7131	247.4778	7	I	4	4023.9458	2012.4765	1341.9868
4329.0933	2165.0503	1443.7026	8	K	3	3910.8617	1955.9345	1304.2921
4476.1617	2238.5845	1492.7254	9	F	2	322.1874	161.5973	108.0673
			10	R	1	175.1190	88.0631	59.0445

[Click] to move table

Linked Peptide + Linker Mass: 3460.58

Linker Mass: 138.06808

ISD TopBP11-766-strep (475) PsfI (63)

```
KDFAPSEK
 |
SDLIPTIKFR
```

KDFAPSEK, MH+ 2248.2223, m/z 562.8110

File: ACE_0704_BT01_StageTip_210min.mzML, Scan: 33941, Exp. m/z: 562.811157226563, Charge: 4

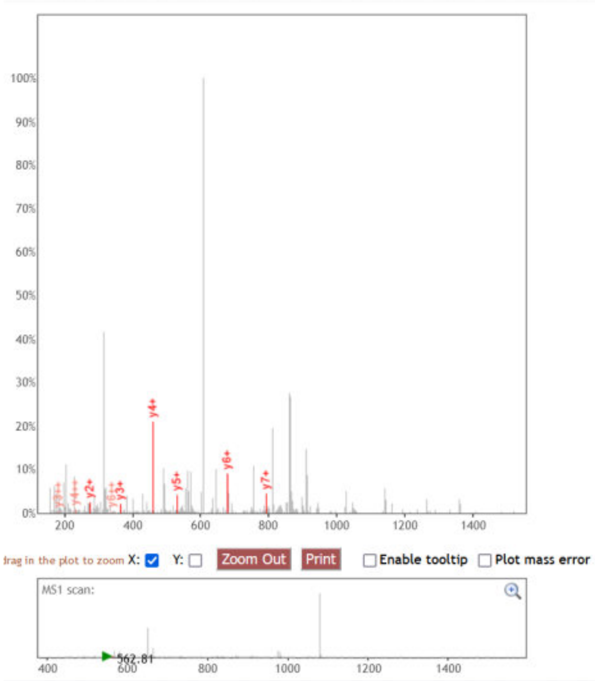

b+	b2+	b3+	#	Seq	#	y+	y2+	y3+
1455.8569	728.4321	485.9571	1	K	8			
1570.8838	785.9456	524.2995	2	D	7	793.3727	397.1900	265.1291
1717.9523	859.4798	573.3223	3	F	6	678.3457	339.6765	226.7868
1788.9894	894.9983	597.0013	4	A	5	531.2773	266.1423	177.7640
1886.0421	943.5247	629.3522	5	P	4	460.2402	230.6237	154.0849
1973.0742	987.0407	658.3629	6	S	3	363.1874	182.0974	121.7340
2102.1168	1051.5620	701.3771	7	E	2	276.1554	138.5813	92.7233
			8	K	1	147.1128	74.0600	49.7091

[Click] to move table

Static Modifications:
C: 57.0215
Linked Peptide + Linker Mass: 1326.75
Linker Mass: 138.068

SDLIPTIKFR, MH+ 2248.2223, m/z 562.8110

File: ACE_0704_BT01_StageTip_210min.mzML, Scan: 33941, Exp. m/z: 562.811157226563, Charge: 4

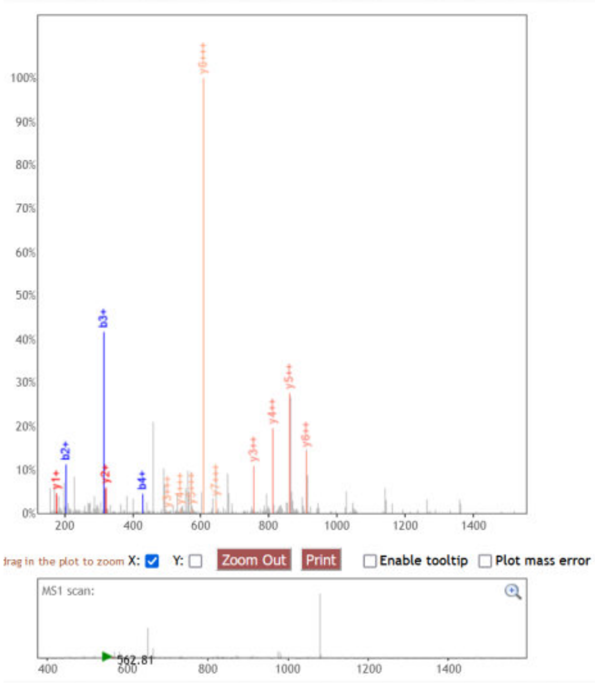

b+	b2+	b3+	#	Seq	#	y+	y2+	y3+
88.0393	44.5233	30.0180	1	S	10			
203.0662	102.0368	68.3603	2	D	9	2161.1903	1081.0988	721.0683
316.1503	158.5788	106.0550	3	L	8	2046.1633	1023.5853	682.7260
429.2344	215.1208	143.7496	4	I	7	1933.0792	967.0433	645.0313
526.2871	263.6472	176.1006	5	P	6	1819.9952	910.5012	607.3366
627.3348	314.1710	209.7831	6	T	5	1722.9424	861.9748	574.9857
740.4189	370.7131	247.4778	7	I	4	1621.8947	811.4510	541.3031
1927.0422	964.0247	643.0189	8	K	3	1508.8107	754.9090	503.6084
2074.1106	1037.5589	692.0417	9	F	2	322.1874	161.5973	108.0673
			10	R	1	175.1190	88.0631	59.0445

[Click] to move table

Static Modifications:
C: 57.0215
Linked Peptide + Linker Mass: 1058.53
Linker Mass: 138.068

ISD TopBP11-766-strep (468) PsfI (63)

KNSSFSK
|
SDLIPTIKFR

KNSSFSK, MH+ 2124.1699, m/z 708.7281

File: ACE_0704_BT01_StageTip_210min.mzML, Scan: 29534, Exp. m/z: 708.728637695313, Charge: 3

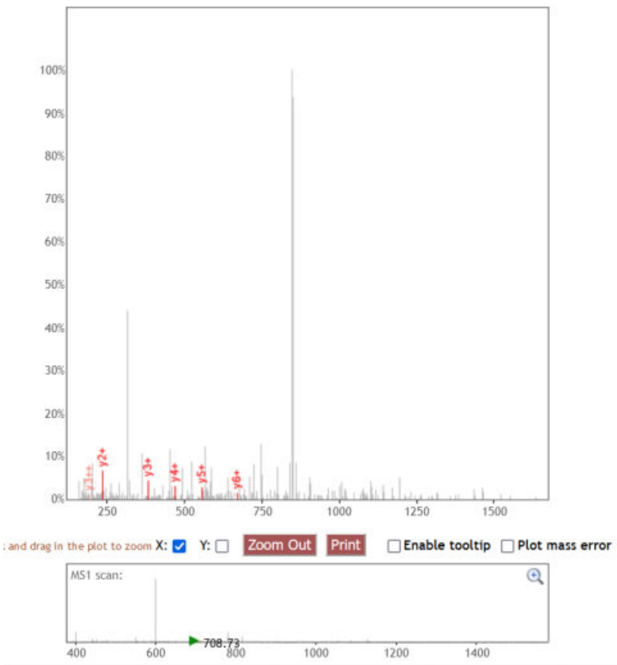

b+	b2+	#	Seq	#	y+	y2+
1455.8569	728.4321	1	K	7		
1569.8998	785.4536	2	N	6	669.3202	335.1638
1656.9319	828.9696	3	S	5	555.2773	278.1423
1743.9639	872.4856	4	S	4	468.2453	234.6263
1891.0323	946.0198	5	F	3	381.2132	191.1103
1978.0643	989.5358	6	S	2	234.1448	117.5761
		7	K	1	147.1128	74.0600

[Click] to move table

Static Modifications:
C: 57.0215
Linked Peptide + Linker Mass: 1326.75
Linker Mass: 138.068

SDLIPTIKFR, MH+ 2124.1699, m/z 708.7281

File: ACE_0704_BT01_StageTip_210min.mzML, Scan: 29534, Exp. m/z: 708.728637695313, Charge: 3

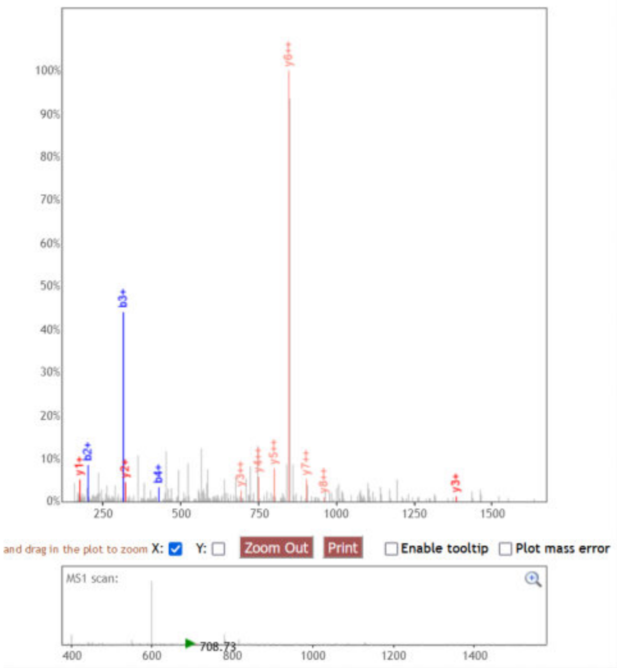

b+	b2+	#	Seq	#	y+	y2+
88.0393	44.5233	1	S	10		
203.0662	102.0368	2	D	9	2037.1378	1019.0725
316.1503	158.5788	3	L	8	1922.1109	961.5591
429.2344	215.1208	4	I	7	1809.0268	905.0170
526.2871	263.6472	5	P	6	1695.9428	848.4750
627.3348	314.1710	6	T	5	1598.8900	799.9486
740.4189	370.7131	7	I	4	1497.8423	749.4248
1802.9898	901.9985	8	K	3	1384.7582	692.8828
1950.0582	975.5327	9	F	2	322.1874	161.5973
		10	R	1	175.1190	88.0631

[Click] to move table

Static Modifications:
C: 57.0215
Linked Peptide + Linker Mass: 934.48
Linker Mass: 138.068

ISD TopBP11-766-strep (466) PsfI (63)

```
     AALLKK
          |
SDLIPTIKFR
```

AALLKK, MH+ 1970.2048, m/z 493.3067

File: ACE_0704_BT01_StageTip_210min.mzML, Scan: 35956, Exp. m/z: 493.306823730469, Charge: 4

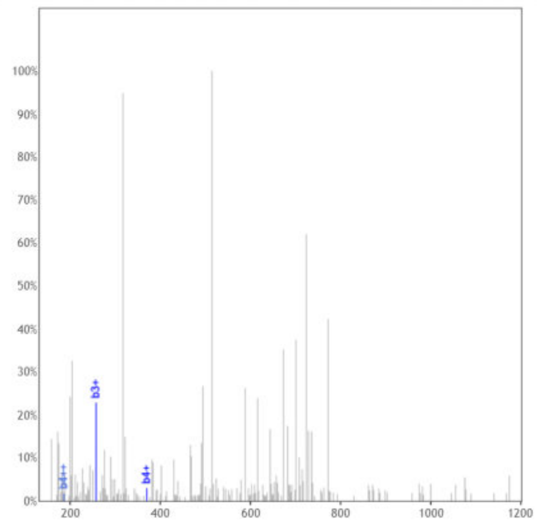

Click and drag in the plot to zoom X: ☑ Y: ☐ Zoom Out Print

☐ Enable tooltip ☐ Plot mass error

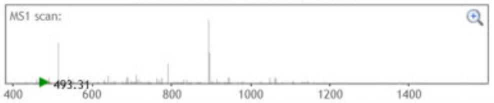

b+	b2+	b3+	#	Seq	#	y+	y2+	y3+
72.0444	36.5258	24.6863	1	A	6			
143.0815	72.0444	48.3654	2	A	5	1899.1677	950.0875	633.7274
256.1656	128.5864	86.0600	3	L	4	1828.1306	914.5689	610.0484
369.2496	185.1285	123.7547	4	L	3	1715.0465	858.0269	572.3537
1824.0993	912.5533	608.7046	5	K	2	1601.9624	801.4849	534.6590
			6	K	1	147.1128	74.0600	49.7091

[Click] to move table

Static Modifications:
C: 57.0215
Linked Peptide + Linker Mass: 1326.75
Linker Mass: 138.068

SDLIPTIKFR, MH+ 1970.2048, m/z 493.3067

File: ACE_0704_BT01_StageTip_210min.mzML, Scan: 35956, Exp. m/z: 493.306823730469, Charge: 4

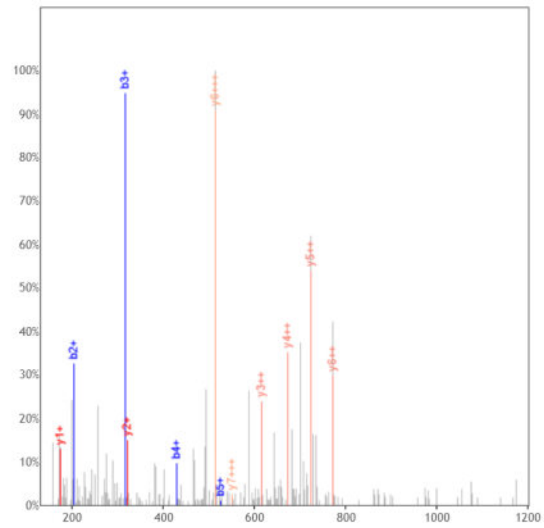

Click and drag in the plot to zoom X: ☑ Y: ☐ Zoom Out Print

☐ Enable tooltip ☐ Plot mass error

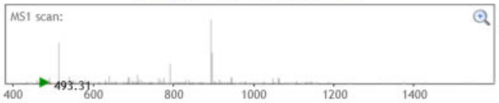

b+	b2+	b3+	#	Seq	#	y+	y2+	y3+
88.0393	44.5233	30.0180	1	S	10			
203.0662	102.0368	68.3603	2	D	9	1883.1728	942.0900	628.3958
316.1503	158.5788	106.0550	3	L	8	1768.1458	884.5765	590.0535
429.2344	215.1208	143.7496	4	I	7	1655.0617	828.0345	552.3588
526.2871	263.6472	176.1006	5	P	6	1541.9777	771.4925	514.6641
627.3348	314.1710	209.7831	6	T	5	1444.9249	722.9661	482.3132
740.4189	370.7131	247.4778	7	I	4	1343.8772	672.4423	448.6306
1649.0247	825.0160	550.3464	8	K	3	1230.7932	615.9002	410.9359
1796.0931	898.5502	599.3692	9	F	2	322.1874	161.5973	108.0673
			10	R	1	175.1190	88.0631	59.0445

[Click] to move table

Static Modifications:
C: 57.0215
Linked Peptide + Linker Mass: 780.51
Linker Mass: 138.068

IGD TopBP11-766-strep (475) PsfIII (80)

```
GLFDNKR
     |
     KDFAPSEK
```

GLFDNKR, MH+ 1907.9861, m/z 477.7520

File: ACE_0704_BT06.mzML, Scan: 10244, Exp. m/z: 477.752380371094, Charge: 4

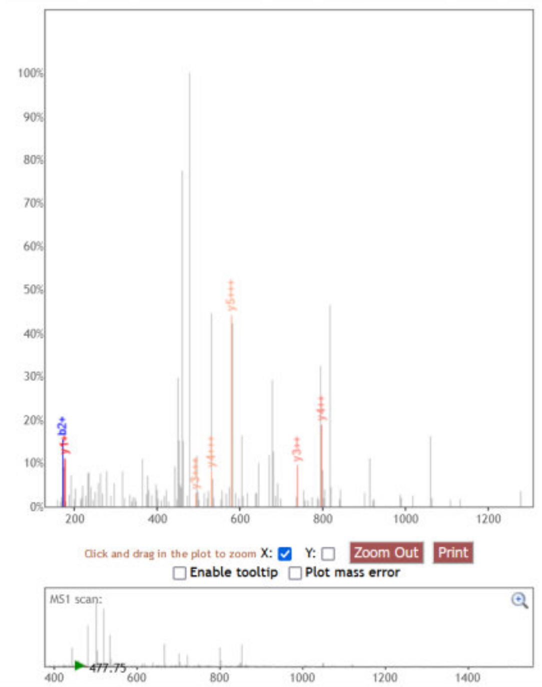

b+	b2+	b3+	#	Seq	#	y+	y2+	y3+
58.0287	29.5180	20.0144	1	G	7			
171.1128	86.0600	57.7091	2	L	6	1850.9646	925.9859	617.6597
318.1812	159.5942	106.7319	3	F	5	1737.8805	869.4439	579.9650
433.2082	217.1077	145.0742	4	D	4	1590.8121	795.9097	530.9422
547.2511	274.1292	183.0885	5	N	3	1475.7852	738.3962	492.5999
1733.8744	867.4408	578.6297	6	K	2	1361.7423	681.3748	454.5856
			7	R	1	175.1190	88.0631	59.0445

[Click] to move table

Static Modifications:
C: 57.0215
Linked Peptide + Linker Mass: 1058.53
Linker Mass: 138.068

KDFAPSEK, MH+ 1907.9861, m/z 477.7520

File: ACE_0704_BT06.mzML, Scan: 10244, Exp. m/z: 477.752380371094, Charge: 4

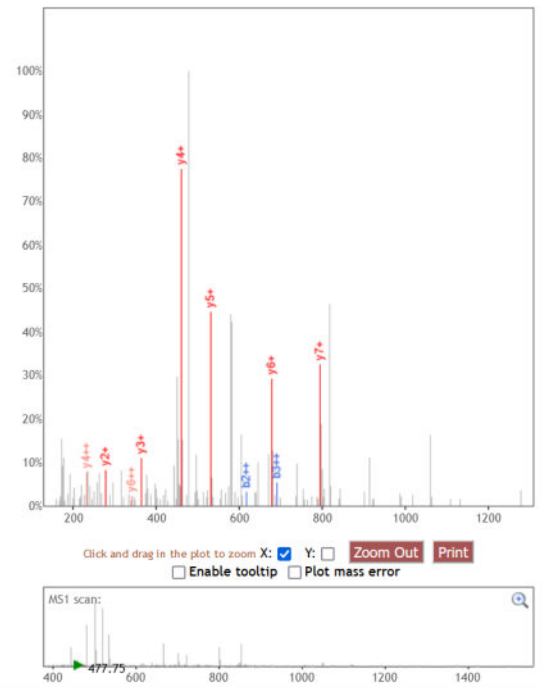

b+	b2+	b3+	#	Seq	#	y+	y2+	y3+
1115.6207	558.3140	372.5451	1	K	8			
1230.6476	615.8275	410.8874	2	D	7	793.3727	397.1900	265.1291
1377.7160	689.3617	459.9102	3	F	6	678.3457	339.6765	226.7868
1448.7532	724.8802	483.5892	4	A	5	531.2773	266.1423	177.7640
1545.8059	773.4066	515.9402	5	P	4	460.2402	230.6237	154.0849
1632.8380	816.9226	544.9508	6	S	3	363.1874	182.0974	121.7340
1761.8805	881.4439	587.9650	7	E	2	276.1554	138.5813	92.7233
			8	K	1	147.1128	74.0600	49.7091

[Click] to move table

Static Modifications:
C: 57.0215
Linked Peptide + Linker Mass: 986.52
Linker Mass: 138.068

IGD TopBP11-766-strep (474) PsfIII (80)

GLFDNKR
|
NSSFSKK

GLFDNKR, MH+ 1783.9336, m/z 595.3161

File: ACE_0704_BT06.mzML, Scan: 9156, Exp. m/z: 595.316101074219, Charge: 3

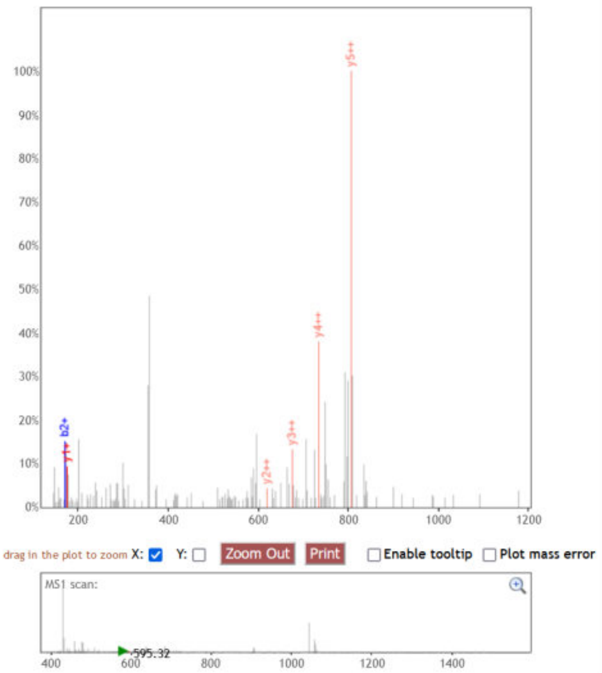

b+	b2+	#	Seq	#	y+	y2+
58.0287	29.5180	1	G	7		
171.1128	86.0600	2	L	6	1726.9122	863.9597
318.1812	159.5942	3	F	5	1613.8281	807.4177
433.2082	217.1077	4	D	4	1466.7597	733.8835
547.2511	274.1292	5	N	3	1351.7328	676.3700
1609.8220	805.4146	6	K	2	1237.6898	619.3486
		7	R	1	175.1190	88.0631

[Click] to move table

Static Modifications:
C: 57.0215
Linked Peptide + Linker Mass: 934.48
Linker Mass: 138.068

NSSFSKK, MH+ 1783.9336, m/z 595.3161

File: ACE_0704_BT06.mzML, Scan: 9156, Exp. m/z: 595.316101074219, Charge: 3

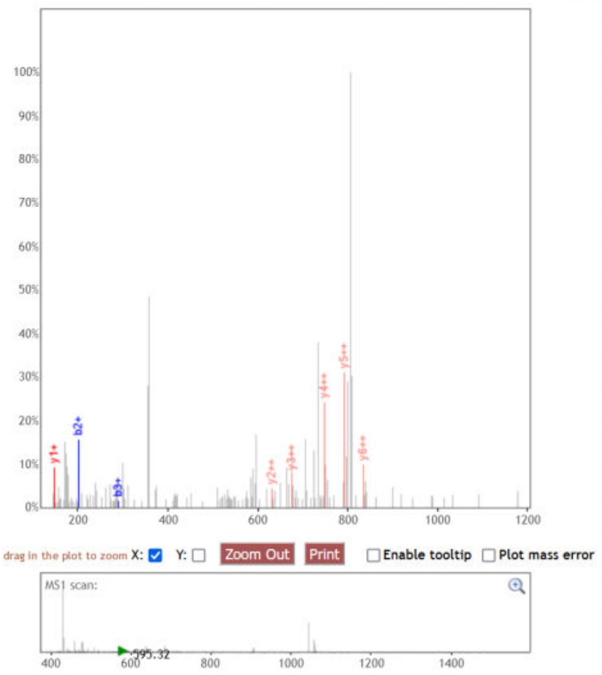

b+	b2+	#	Seq	#	y+	y2+
115.0502	58.0287	1	N	7		
202.0822	101.5448	2	S	6	1669.8907	835.4490
289.1143	145.0608	3	S	5	1582.8587	791.9330
436.1827	218.5950	4	F	4	1495.8267	748.4170
523.2147	262.1110	5	S	3	1348.7582	674.8828
1637.8281	819.4177	6	K	2	1261.7262	631.3667
		7	K	1	147.1128	74.0600

[Click] to move table

Static Modifications:
C: 57.0215
Linked Peptide + Linker Mass: 986.52
Linker Mass: 138.068

IGD TopBP11-766-strep (473) PsfIII (80)

```
GLFDNKR
     |
 NSSFSKK
```

GLFDNKR, MH+ 1783.9336, m/z 595.3161

File: ACE_0704_BT05.mzML, Scan: 9151, Exp. m/z: 595.316223144531, Charge: 3

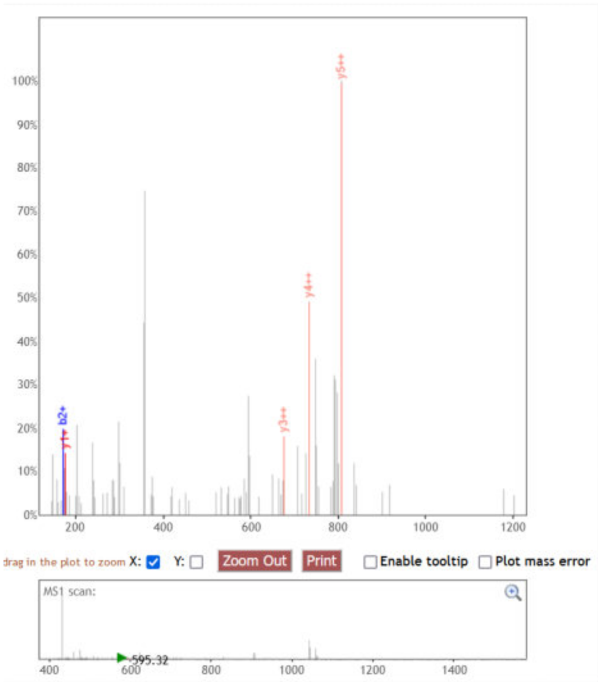

b+	b2+	#	Seq	#	y+	y2+
58.0287	29.5180	1	G	7		
171.1128	86.0600	2	L	6	1726.9122	863.9597
318.1812	159.5942	3	F	5	1613.8281	807.4177
433.2082	217.1077	4	D	4	1466.7597	733.8835
547.2511	274.1292	5	N	3	1351.7328	676.3700
1609.8220	805.4146	6	K	2	1237.6898	619.3486
		7	R	1	175.1190	88.0631

[Click] to move table

Static Modifications:
C: 57.0215
Linked Peptide + Linker Mass: 934.48
Linker Mass: 138.068

NSSFSKK, MH+ 1783.9336, m/z 595.3161

File: ACE_0704_BT05.mzML, Scan: 9151, Exp. m/z: 595.316223144531, Charge: 3

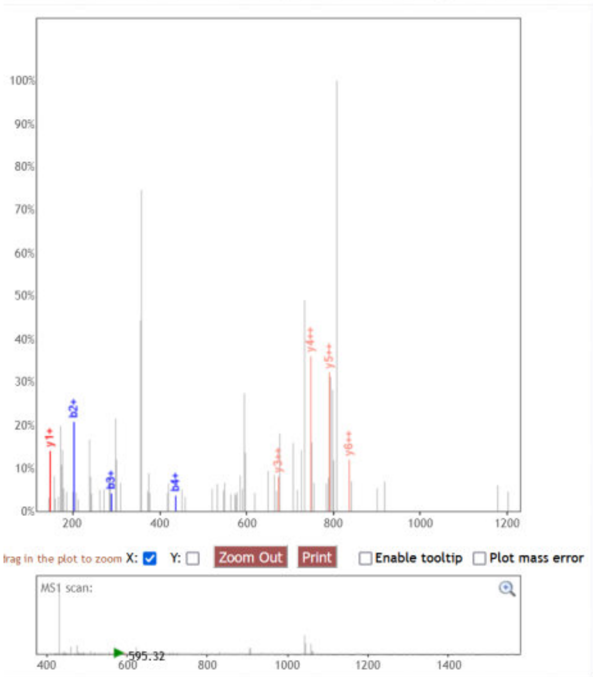

b+	b2+	#	Seq	#	y+	y2+
115.0502	58.0287	1	N	7		
202.0822	101.5448	2	S	6	1669.8907	835.4490
289.1143	145.0608	3	S	5	1582.8587	791.9330
436.1827	218.5950	4	F	4	1495.8267	748.4170
1509.7332	755.3702	5	S	3	1348.7582	674.8828
1637.8281	819.4177	6	K	2	275.2078	138.1075
		7	K	1	147.1128	74.0600

[Click] to move table

Static Modifications:
C: 57.0215
Linked Peptide + Linker Mass: 986.52
Linker Mass: 138.068

IGD TopBP11-766-strep (468) PsfIII (80)

```
GLFDNKR
    |
    KNSSFSK
```

GLFDNKR, MH+ 1783.9336, m/z 595.3161

File: ACE_0704_BT05.mzML, Scan: 8482, Exp. m/z: 595.315673828125, Charge: 3

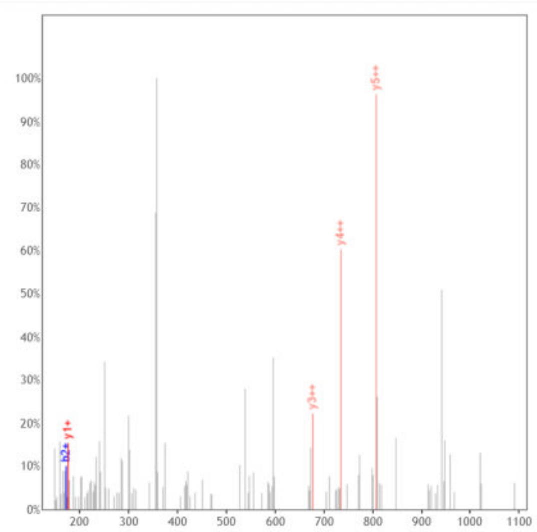

d drag in the plot to zoom X: ☑ Y: ☐ Zoom Out Print ☐ Enable tooltip ☐ Plot mass error

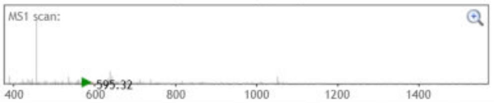

b+	b2+	#	Seq	#	y+	y2+
58.0287	29.5180	1	G	7		
171.1128	86.0600	2	L	6	1726.9122	863.9597
318.1812	159.5942	3	F	5	1613.8281	807.4177
433.2082	217.1077	4	D	4	1466.7597	733.8835
547.2511	274.1292	5	N	3	1351.7328	676.3700
1609.8220	805.4146	6	K	2	1237.6898	619.3486
		7	R	1	175.1190	88.0631

[Click] to move table

Static Modifications:
C: 57.0215
Linked Peptide + Linker Mass: 934.48
Linker Mass: 138.068

KNSSFSK, MH+ 1783.9336, m/z 595.3161

File: ACE_0704_BT05.mzML, Scan: 8482, Exp. m/z: 595.315673828125, Charge: 3

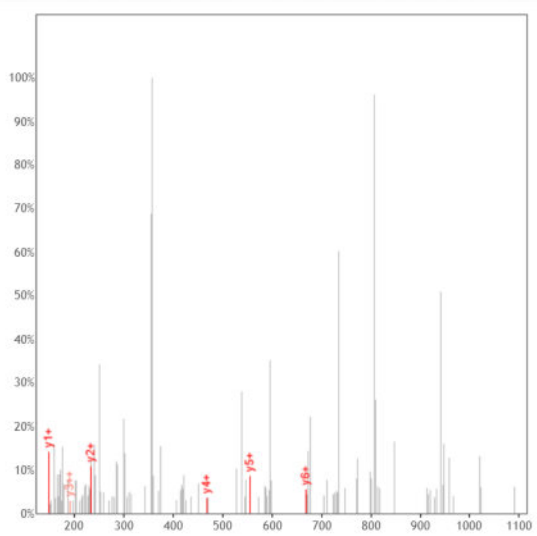

drag in the plot to zoom X: ☑ Y: ☐ Zoom Out Print ☐ Enable tooltip ☐ Plot mass error

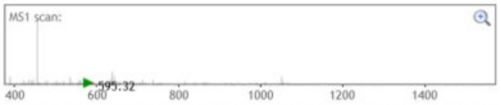

b+	b2+	#	Seq	#	y+	y2+
1115.6207	558.3140	1	K	7		
1229.6636	615.3354	2	N	6	669.3202	335.1638
1316.6956	658.8515	3	S	5	555.2773	278.1423
1403.7277	702.3675	4	S	4	468.2453	234.6263
1550.7961	775.9017	5	F	3	381.2132	191.1103
1637.8281	819.4177	6	S	2	234.1448	117.5761
		7	K	1	147.1128	74.0600

[Click] to move table

Static Modifications:
C: 57.0215
Linked Peptide + Linker Mass: 986.52
Linker Mass: 138.068

IGD TopBP11-766-strep (466) PsfIII (80)

```
 AALLKK
      |
GLFDNKR
```

AALLKK, MH+ 1629.9686, m/z 543.9944

File: ACE_0704_BT05.mzML, Scan: 10736, Exp. m/z: 543.994750976563, Charge: 3

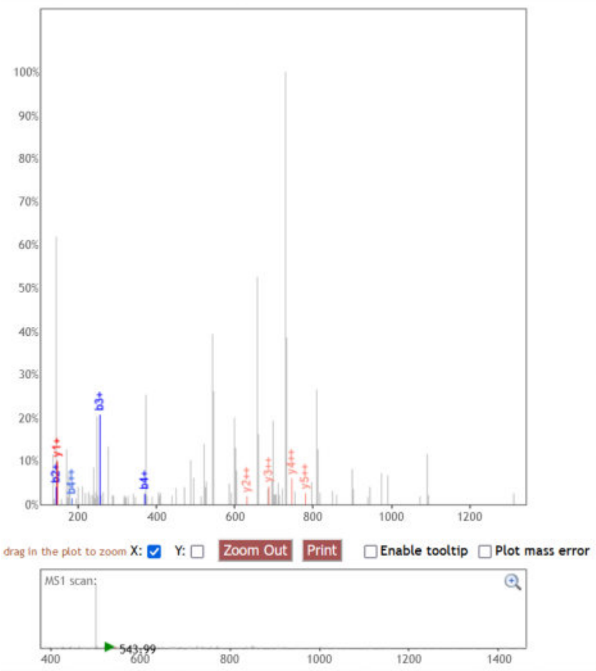

b+	b2+	#	Seq	#	y+	y2+
72.0444	36.5258	1	A	6		
143.0815	72.0444	2	A	5	1558.9315	779.9694
256.1656	128.5864	3	L	4	1487.8943	744.4508
369.2496	185.1285	4	L	3	1374.8103	687.9088
1483.8630	742.4352	5	K	2	1261.7262	631.3667
		6	K	1	147.1128	74.0600

[Click] to move table

Static Modifications:
C: 57.0215
Linked Peptide + Linker Mass: 986.52
Linker Mass: 138.068

GLFDNKR, MH+ 1629.9686, m/z 543.9944

File: ACE_0704_BT05.mzML, Scan: 10736, Exp. m/z: 543.994750976563, Charge: 3

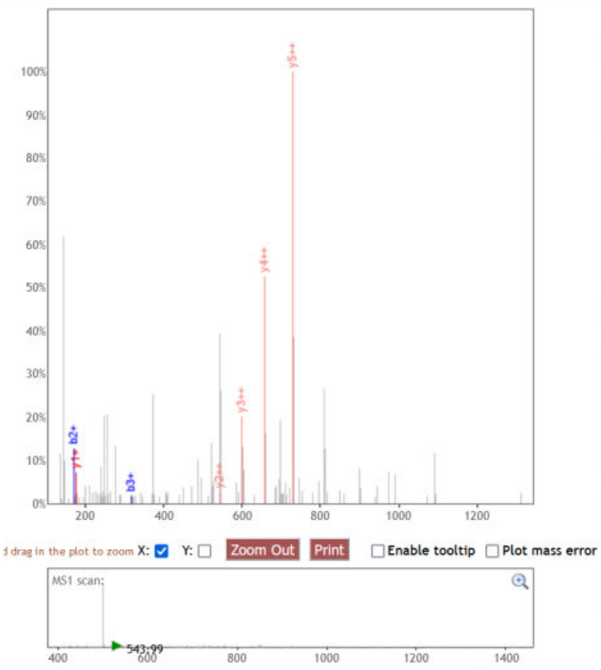

b+	b2+	#	Seq	#	y+	y2+
58.0287	29.5180	1	G	7		
171.1128	86.0600	2	L	6	1572.9471	786.9772
318.1812	159.5942	3	F	5	1459.8630	730.4352
433.2082	217.1077	4	D	4	1312.7946	656.9010
547.2511	274.1292	5	N	3	1197.7677	599.3875
1455.8569	728.4321	6	K	2	1083.7248	542.3660
		7	R	1	175.1190	88.0631

[Click] to move table

Static Modifications:
C: 57.0215
Linked Peptide + Linker Mass: 780.51
Linker Mass: 138.068

ISD TopBP11-766-strep (480) PsfIII (80)

```
 DFAPSEK
      |
GLFDNKR
```

DFAPSEK, MH+ 1779.8911, m/z 593.9686

File: ACE_0704_BT01_StageTip_210min.mzML, Scan: 22561, Exp. m/z: 593.968200683594, Charge: 3

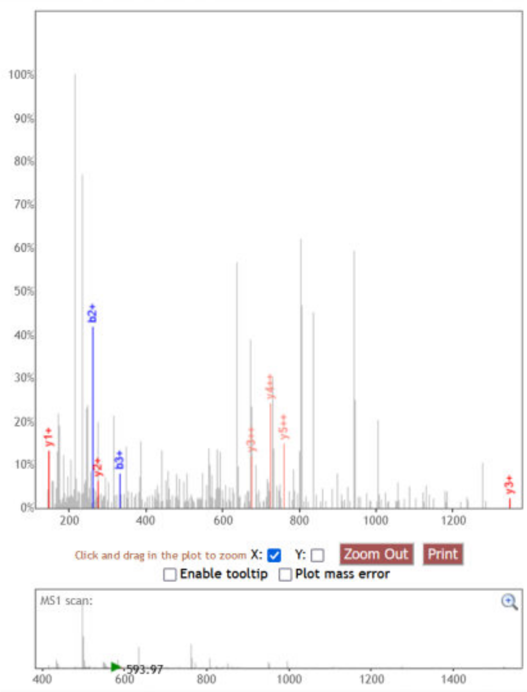

b+	b2+	#	Seq	#	y+	y2+
116.0342	58.5207	1	D	7		
263.1026	132.0550	2	F	6	1664.8642	832.9357
334.1397	167.5735	3	A	5	1517.7958	759.4015
431.1925	216.0999	4	P	4	1446.7586	723.8830
1504.7430	752.8751	5	S	3	1349.7059	675.3566
1633.7856	817.3964	6	E	2	276.1554	138.5813
		7	K	1	147.1128	74.0600

[Click] to move table

Static Modifications:
C: 57.0215
Linked Peptide + Linker Mass: 986.52
Linker Mass: 138.068

GLFDNKR, MH+ 1779.8911, m/z 593.9686

File: ACE_0704_BT01_StageTip_210min.mzML, Scan: 22561, Exp. m/z: 593.968200683594, Charge: 3

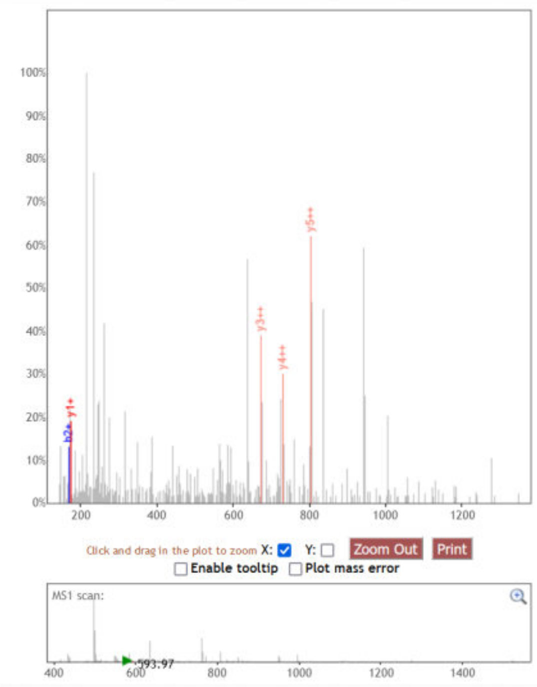

b+	b2+	#	Seq	#	y+	y2+
58.0287	29.5180	1	G	7		
171.1128	86.0600	2	L	6	1722.8696	861.9385
318.1812	159.5942	3	F	5	1609.7856	805.3964
433.2082	217.1077	4	D	4	1462.7172	731.8622
547.2511	274.1292	5	N	3	1347.6902	674.3488
1605.7794	803.3934	6	K	2	1233.6473	617.3273
		7	R	1	175.1190	88.0631

[Click] to move table

Static Modifications:
C: 57.0215
Linked Peptide + Linker Mass: 930.43
Linker Mass: 138.068

ISD TopBP11-766-strep (475) PsfIII (80)

```
GLFDNKR
         |
         KDFAPSEK
```

GLFDNKR, MH+ 1907.9861, m/z 636.6669

File: ACE_0704_BT01_StageTip_210min.mzML, Scan: 21105, Exp. m/z: 636.666381835938, Charge: 3

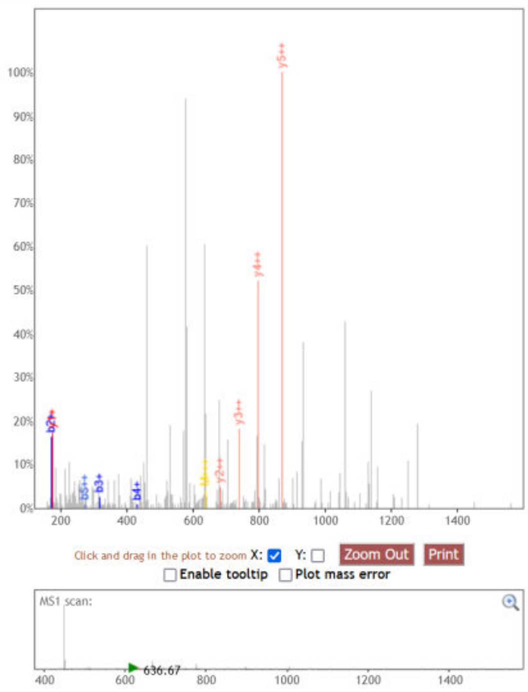

b+	b2+	#	Seq	#	y+	y2+
58.0287	29.5180	1	G	7		
171.1128	86.0600	2	L	6	1850.9646	925.9859
318.1812	159.5942	3	F	5	1737.8805	869.4439
433.2082	217.1077	4	D	4	1590.8121	795.9097
547.2511	274.1292	5	N	3	1475.7852	738.3962
1733.8744	867.4408	6	K	2	1361.7423	681.3748
		7	R	1	175.1190	88.0631

[Click] to move table

Static Modifications:
C: 57.0215
Linked Peptide + Linker Mass: 1058.53
Linker Mass: 138.068

KDFAPSEK, MH+ 1907.9861, m/z 636.6669

File: ACE_0704_BT01_StageTip_210min.mzML, Scan: 21105, Exp. m/z: 636.666381835938, Charge: 3

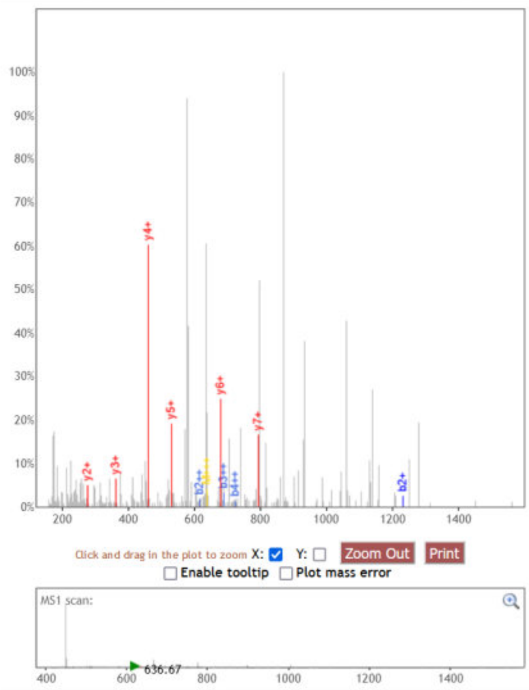

b+	b2+	#	Seq	#	y+	y2+
1115.6207	558.3140	1	K	8		
1230.6476	615.8275	2	D	7	793.3727	397.1900
1377.7160	689.3617	3	F	6	678.3457	339.6765
1448.7532	724.8802	4	A	5	531.2773	266.1423
1545.8059	773.4066	5	P	4	460.2402	230.6237
1632.8380	816.9226	6	S	3	363.1874	182.0974
1761.8805	881.4439	7	E	2	276.1554	138.5813
		8	K	1	147.1128	74.0600

[Click] to move table

Static Modifications:
C: 57.0215
Linked Peptide + Linker Mass: 986.52
Linker Mass: 138.068

ISD TopBP11-766-strep (474) PsfIII (80)

GLFDNKR
|
NSSFSKK

GLFDNKR, MH+ 1783.9336, m/z 595.3161

File: ACE_0704_BT01_StageTip_210min.mzML, Scan: 17827, Exp. m/z: 595.31591796875, Charge: 3

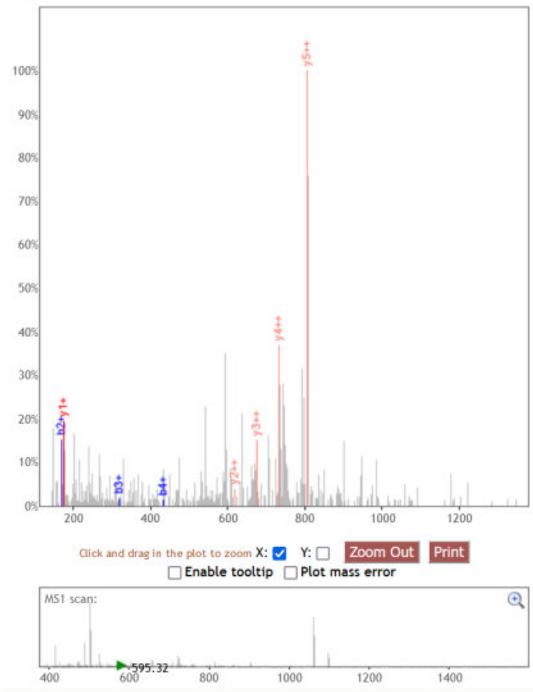

b+	b2+	#	Seq	#	y+	y2+
58.0287	29.5180	1	G	7		
171.1128	86.0600	2	L	6	1726.9122	863.9597
318.1812	159.5942	3	F	5	1613.8281	807.4177
433.2082	217.1077	4	D	4	1466.7597	733.8835
547.2511	274.1292	5	N	3	1351.7328	676.3700
1609.8220	805.4146	6	K	2	1237.6898	619.3486
		7	R	1	175.1190	88.0631

[Click] to move table

Static Modifications:
C: 57.0215
Linked Peptide + Linker Mass: 934.48
Linker Mass: 138.068

NSSFSKK, MH+ 1783.9336, m/z 595.3161

File: ACE_0704_BT01_StageTip_210min.mzML, Scan: 17827, Exp. m/z: 595.31591796875, Charge: 3

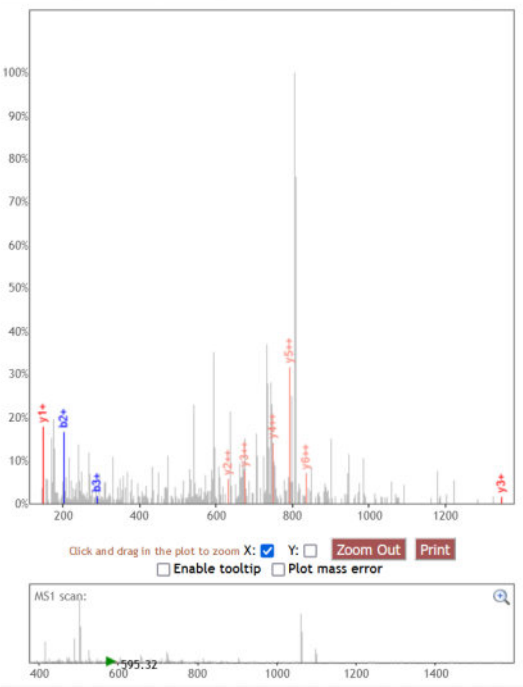

b+	b2+	#	Seq	#	y+	y2+
115.0502	58.0287	1	N	7		
202.0822	101.5448	2	S	6	1669.8907	835.4490
289.1143	145.0608	3	S	5	1582.8587	791.9330
436.1827	218.5950	4	F	4	1495.8267	748.4170
523.2147	262.1110	5	S	3	1348.7582	674.8828
1637.8281	819.4177	6	K	2	1261.7262	631.3667
		7	K	1	147.1128	74.0600

[Click] to move table

Static Modifications:
C: 57.0215
Linked Peptide + Linker Mass: 986.52
Linker Mass: 138.068

ISD TopBP11-766-strep (468) PsfIII (80)

```
GLFDNKR
    |
    KNSSFSK
```

GLFDNKR, MH+ 1783.9336, m/z 446.7389

File: ACE_0704_BT01_StageTip_210min.mzML, Scan: 16316, Exp. m/z: 446.738952636719, Charge: 4

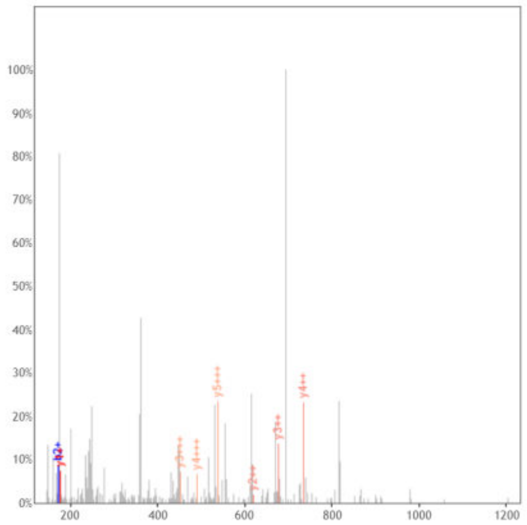

Click and drag in the plot to zoom X: ☑ Y: ☐ Zoom Out Print

☐ Enable tooltip ☐ Plot mass error

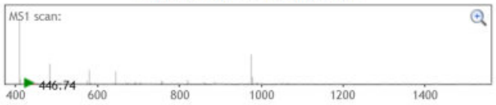

b+	b2+	b3+	#	Seq	#	y+	y2+	y3+
58.0287	29.5180	20.0144	1	G	7			
171.1128	86.0600	57.7091	2	L	6	1726.9122	863.9597	576.3089
318.1812	159.5942	106.7319	3	F	5	1613.8281	807.4177	538.6142
433.2082	217.1077	145.0742	4	D	4	1466.7597	733.8835	489.5914
547.2511	274.1292	183.0885	5	N	3	1351.7328	676.3700	451.2491
1609.8220	805.4146	537.2788	6	K	2	1237.6898	619.3486	413.2348
			7	R	1	175.1190	88.0631	59.0445

[Click] to move table

Static Modifications:
C: 57.0215
Linked Peptide + Linker Mass: 934.48
Linker Mass: 138.068

KNSSFSK, MH+ 1783.9336, m/z 446.7389

File: ACE_0704_BT01_StageTip_210min.mzML, Scan: 16316, Exp. m/z: 446.738952636719, Charge: 4

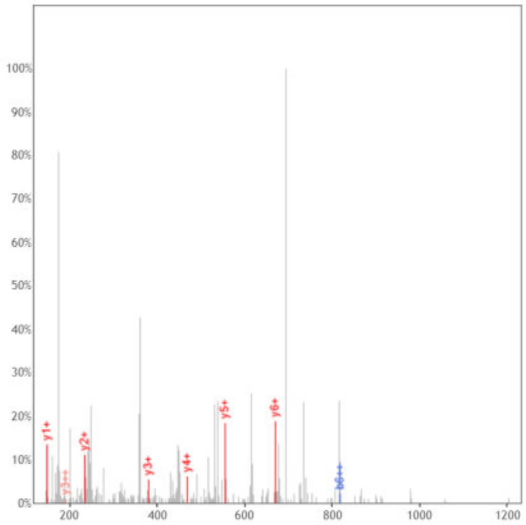

Click and drag in the plot to zoom X: ☑ Y: ☐ Zoom Out Print

☐ Enable tooltip ☐ Plot mass error

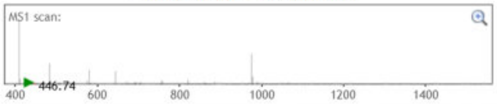

b+	b2+	b3+	#	Seq	#	y+	y2+	y3+
1115.6207	558.3140	372.5451	1	K	7			
1229.6636	615.3354	410.5594	2	N	6	669.3202	335.1638	223.7783
1316.6956	658.8515	439.5701	3	S	5	555.2773	278.1423	185.7640
1403.7277	702.3675	468.5807	4	S	4	468.2453	234.6263	156.7533
1550.7961	775.9017	517.6035	5	F	3	381.2132	191.1103	127.7426
1637.8281	819.4177	546.6142	6	S	2	234.1448	117.5761	78.7198
			7	K	1	147.1128	74.0600	49.7091

[Click] to move table

Static Modifications:
C: 57.0215
Linked Peptide + Linker Mass: 986.52
Linker Mass: 138.068

ISD   TopBP11-766-strep (466) PsfIII (80)

```
 AALLKK
     |
GLFDNKR
```

AALLKK, MH+ 1629.9686, m/z 543.9944

File: ACE_0704_BT01_StageTip_210min.mzML, Scan: 21980, Exp. m/z: 543.994140625, Charge: 3

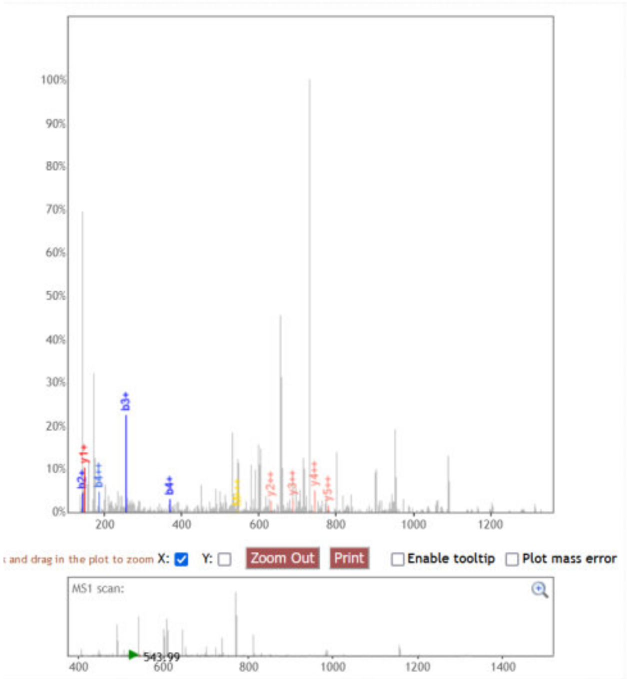

b+	b2+	#	Seq	#	y+	y2+
72.0444	36.5258	1	A	6		
143.0815	72.0444	2	A	5	1558.9315	779.9694
256.1656	128.5864	3	L	4	1487.8943	744.4508
369.2496	185.1285	4	L	3	1374.8103	687.9088
1483.8630	742.4352	5	K	2	1261.7262	631.3667
		6	K	1	147.1128	74.0600

[Click] to move table

Static Modifications:
C: 57.0215
Linked Peptide + Linker Mass: 986.52
Linker Mass: 138.068

GLFDNKR, MH+ 1629.9686, m/z 543.9944

File: ACE_0704_BT01_StageTip_210min.mzML, Scan: 21980, Exp. m/z: 543.994140625, Charge: 3

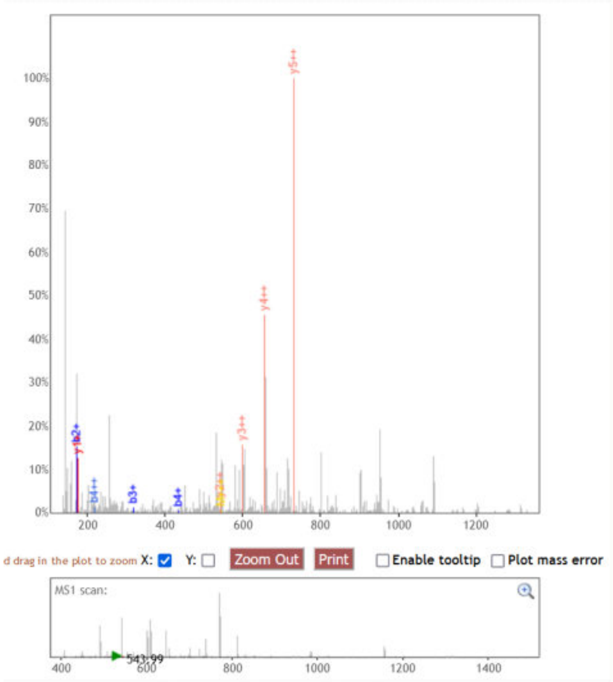

b+	b2+	#	Seq	#	y+	y2+
58.0287	29.5180	1	G	7		
171.1128	86.0600	2	L	6	1572.9471	786.9772
318.1812	159.5942	3	F	5	1459.8630	730.4352
433.2082	217.1077	4	D	4	1312.7946	656.9010
547.2511	274.1292	5	N	3	1197.7677	599.3875
1455.8569	728.4321	6	K	2	1083.7248	542.3660
		7	R	1	175.1190	88.0631

[Click] to move table

Static Modifications:
C: 57.0215
Linked Peptide + Linker Mass: 780.51
Linker Mass: 138.068

ISD TopBP11-766-strep (480) PsfIII (74)

```
        DFAPSEKHEQADEDLLSQYENGSSTVVEAK
             |
LELPLWLAKGLFDNK
```

DFAPSEKHEQADEDLLSQYENGSSTVVEAK, MH+ 5217.5790, m/z 1044.3216

File: ACE_0704_BT01_StageTip_210min-calib.mzML, Scan: 57130, Exp. m/z: 1045.1219288370896, Charge: 5

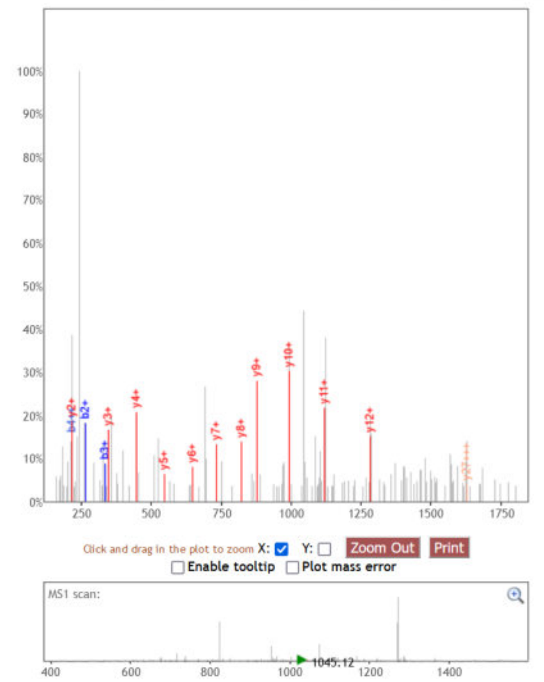

b+	b2+	b3+	#	Seq	#	y+	y2+	y3+
116.0342	58.5207	39.3496	1	D	30			
263.1026	132.0550	88.3724	2	F	29	5102.5521	2551.7797	1701.5222
334.1397	167.5735	112.0514	3	A	28	4955.4837	2478.2455	1652.4994
431.1925	216.0999	144.4024	4	P	27	4884.4466	2442.7269	1628.8204
2412.2850	1206.6461	804.7665	5	S	26	4787.3938	2394.2005	1596.4695
2541.3276	1271.1674	847.7807	6	E	25	2806.3014	1403.6543	936.1053
2669.4225	1335.2149	890.4790	7	K	24	2677.2588	1339.1330	893.0911
2806.4814	1403.7444	936.1653	8	H	23	2549.1638	1275.0855	850.3928
2935.5240	1468.2656	979.1795	9	E	22	2412.1049	1206.5561	804.7065
3063.5826	1532.2949	1021.8657	10	Q	21	2283.0623	1142.0348	761.6923
3134.6197	1567.8135	1045.5448	11	A	20	2155.0037	1078.0055	719.0061
3249.6467	1625.3270	1083.8871	12	D	19	2083.9666	1042.4869	695.3271
3378.6893	1689.8483	1126.9013	13	E	18	1968.9397	984.9735	656.9847
3493.7162	1747.3617	1165.2436	14	D	17	1839.8971	920.4522	613.9705
3606.8003	1803.9038	1202.9383	15	L	16	1724.8701	862.9387	575.6282
3719.8843	1860.4458	1240.6330	16	L	15	1611.7861	806.3967	537.9335
3806.9164	1903.9618	1269.6436	17	S	14	1498.7020	749.8546	500.2388
3934.9749	1967.9911	1312.3298	18	Q	13	1411.6700	706.3386	471.2282
4098.0383	2049.5228	1366.6843	19	Y	12	1283.6114	642.3093	428.5420
4227.0808	2114.0441	1409.6985	20	E	11	1120.5481	560.7777	374.1875
4341.1238	2171.0655	1447.7128	21	N	10	991.5055	496.2564	331.1733
4398.1452	2199.5763	1466.7199	22	G	9	877.4625	439.2349	293.1590
4485.1773	2243.0923	1495.7306	23	S	8	820.4411	410.7242	274.1519
4572.2093	2286.6083	1524.7413	24	S	7	733.4090	367.2082	245.1412
4673.2570	2337.1321	1558.4238	25	T	6	646.3770	323.6921	216.1305
4772.3254	2386.6663	1591.4466	26	V	5	545.3293	273.1683	182.4480
4871.3938	2436.2005	1624.4695	27	V	4	446.2609	223.6341	149.4252
5000.4364	2500.7218	1667.4836	28	E	3	347.1925	174.0999	116.4024
5071.4735	2536.2404	1691.1627	29	A	2	218.1499	109.5786	73.3882
			30	K	1	147.1128	74.0600	49.7091

[Click] to move table

Linked Peptide + Linker Mass: 1894.06
Linker Mass: 138.06808

LELPLWLAKGLFDNK, MH+ 5217.5790, m/z 1044.3216

File: ACE_0704_BT01_StageTip_210min-calib.mzML, Scan: 57130, Exp. m/z: 1045.1219288370896, Charge: 5

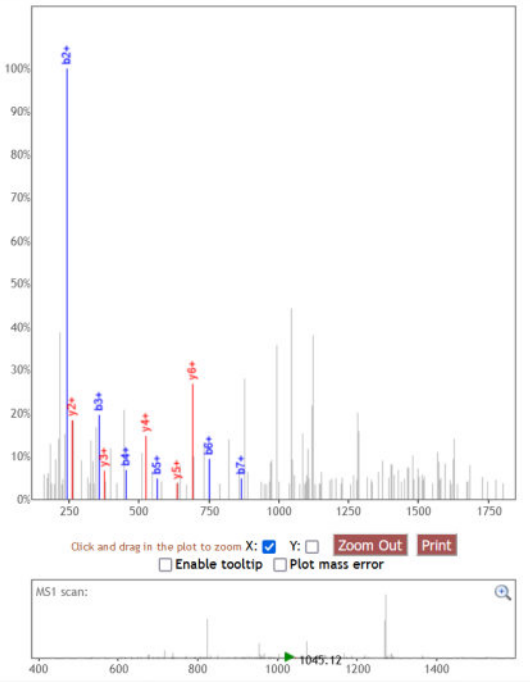

b+	b2+	b3+	#	Seq	#	y+	y2+	y3+
114.0913	57.5493	38.7020	1	L	15			
243.1339	122.0706	81.7162	2	E	14	5104.4950	2552.7511	1702.1698
356.2180	178.6126	119.4108	3	L	13	4975.4524	2488.2298	1659.1556
453.2708	227.1390	151.7618	4	P	12	4862.3683	2431.6878	1621.4610
566.3548	283.6811	189.4565	5	L	11	4765.3156	2383.1614	1589.1100
752.4341	376.7207	251.4829	6	W	10	4652.2315	2326.6194	1551.4153
865.5182	433.2627	289.1776	7	L	9	4466.1522	2233.5797	1489.3889
936.5553	468.7813	312.8566	8	A	8	4353.0681	2177.0377	1451.6942
4525.2297	2263.1185	1509.0814	9	K	7	4282.0310	2141.5191	1428.0152
4582.2512	2291.6292	1528.0886	10	G	6	693.3566	347.1819	231.7904
4695.3352	2348.1713	1565.7833	11	L	5	636.3352	318.6712	212.7832
4842.4036	2421.7055	1614.8061	12	F	4	523.2511	262.1292	175.0885
4957.4306	2479.2189	1653.1484	13	D	3	376.1827	188.5950	126.0657
5071.4735	2536.2404	1691.1627	14	N	2	261.1557	131.0815	87.7234
			15	K	1	147.1128	74.0600	49.7091

[Click] to move table

Linked Peptide + Linker Mass: 3460.58
Linker Mass: 138.06808
