# Supplementary material for: TopBP1 utilises a bipartite GINS binding mode to support genome replication
Source: Nat Commun. 2024 Feb 27;15:1797. doi: 10.1038/s41467-024-45946-0 (PMC10899662; doi:10.1038/s41467-024-45946-0)
Supplement: Supplementary file 7 — Reporting Summary [file 41467_2024_45946_MOESM7_ESM.pdf]

nature portfolio

Corresponding author(s): Dominik Boos, Matthew Day, Antony W Oliver, Laurence H Pearl

Last updated by author(s): Jan 18, 2024

Reporting Summary

Nature Portfolio wishes to improve the reproducibility of the work that we publish. This form provides structure for consistency and transparency in reporting. For further information on Nature Portfolio policies, see our Editorial Policies and the Editorial Policy Checklist.

Statistics

For all statistical analyses, confirm that the following items are present in the figure legend, table legend, main text, or Methods section.

n/a	Confirmed	
☐	☒	The exact sample size (*n*) for each experimental group/condition, given as a discrete number and unit of measurement
☐	☒	A statement on whether measurements were taken from distinct samples or whether the same sample was measured repeatedly
☐	☒	The statistical test(s) used AND whether they are one- or two-sided<br>*Only common tests should be described solely by name; describe more complex techniques in the Methods section.*
☒	☐	A description of all covariates tested
☒	☐	A description of any assumptions or corrections, such as tests of normality and adjustment for multiple comparisons
☐	☒	A full description of the statistical parameters including central tendency (e.g. means) or other basic estimates (e.g. regression coefficient) AND variation (e.g. standard deviation) or associated estimates of uncertainty (e.g. confidence intervals)
☐	☒	For null hypothesis testing, the test statistic (e.g. *F*, *t*, *r*) with confidence intervals, effect sizes, degrees of freedom and *P* value noted<br>*Give P values as exact values whenever suitable.*
☒	☐	For Bayesian analysis, information on the choice of priors and Markov chain Monte Carlo settings
☒	☐	For hierarchical and complex designs, identification of the appropriate level for tests and full reporting of outcomes
☒	☐	Estimates of effect sizes (e.g. Cohen's *d*, Pearson's *r*), indicating how they were calculated

Our web collection on statistics for biologists contains articles on many of the points above.

Software and code

Policy information about availability of computer code

Data collection	Cluspro, MDockPep, HPEPDOCK, FlexPepDock, Colab Alphafold advanced (initiatial Psf1-TopBP1 model), AI600 Imaging system (GE helthcare); Alphafold Multimer version 2.2.0; Cytiva UNICORN 5.3.1; Orbitrap Fusion Lumos Tune Application (3.3.2782.28); Xcalibur (4.3.73.11); MaxQuant (v2.0.3.0); MetaMorpheus (0.0.320); pLink2 (2.3.9); EPU (Thermo Fisher Scientific); cryoSPARC; Coot, Phenix
Data analysis	Adobe Illustrator 2020 26.0.2 and 2022 v24.3; Chimera X, Adobe Photoshop 2020 21.2.3 and 2022 v24.1.0, ImageJ 1.51j8; ProXL: metaMorph2ProxlXML.jar; plink2toProxlXML.jar; Microsoft Excel (16.68); RELION4.0; MOTIONCORE2 implemented in RELION4.0; Alphapickle 1.5.4

For manuscripts utilizing custom algorithms or software that are central to the research but not yet described in published literature, software must be made available to editors and reviewers. We strongly encourage code deposition in a community repository (e.g. GitHub). See the Nature Portfolio guidelines for submitting code & software for further information.

Data

Policy information about availability of data

All manuscripts must include a data availability statement. This statement should provide the following information, where applicable:

- Accession codes, unique identifiers, or web links for publicly available datasets
- A description of any restrictions on data availability
- For clinical datasets or third party data, please ensure that the statement adheres to our policy

The mass spectrometry proteomics data for the proximity dependent biotinylation experiments, the cross-linking experiments and the chromatin mass spectrometry (CHROMASS) experiments have been deposited to the ProteomeXchange Consortium via the PRIDE88 partner repository (https://www.ebi.ac.uk/pride/archive/) with the dataset identifiers PXD040000 (PDB experiments), PXD040156 (CL-MS experiments) and PXD040024 (CHROMASS experiments). During the review process the data can be accessed via the following Reviewer accounts:
PXD040000: (Username: reviewer_pxd040000@ebi.ac.uk; Password: Lk9VLXVF)
PXD040156: (Username: reviewer_pxd040156@ebi.ac.uk; Password: NLNLORQl)
PXD040024: (Username: reviewer_pxd040024@ebi.ac.uk; Password: 5OGweuE6)

Cryo-EM maps and refined coordinates have been deposited in the EMDB and Protein Databank as EMD- 16916 and PDB ID 8OK2 respectively.

Research involving human participants, their data, or biological material

Policy information about studies with human participants or human data. See also policy information about sex, gender (identity/presentation), and sexual orientation and race, ethnicity and racism.

Reporting on sex and gender	N/A
Reporting on race, ethnicity, or other socially relevant groupings	N/A
Population characteristics	N/A
Recruitment	N/A
Ethics oversight	N/A

Note that full information on the approval of the study protocol must also be provided in the manuscript.

Field-specific reporting

Please select the one below that is the best fit for your research. If you are not sure, read the appropriate sections before making your selection.

☒ Life sciences ☐ Behavioural & social sciences ☐ Ecological, evolutionary & environmental sciences

For a reference copy of the document with all sections, see nature.com/documents/nr-reporting-summary-flat.pdf

Life sciences study design

All studies must disclose on these points even when the disclosure is negative.

Sample size	Replication experiments averaging DNA synthesis values (incorpration of radioactive nucleotide) were done based on three biological replicates. The chromass experiment used four independent bioloigical replicates. APEX2-based proximity biotinylation experiments used four biological replicates. No sample size calculation was performed. Sample size was chosen in order to allow statistically sufficient conclusions.
Data exclusions	none
Replication	DNA replication experiments using Xenopus egg extracts in the main figures were done were done with three independent biological replicates, and subsequent quantifications were statistically analysed using standard error of the mean. Two independent anti-TopBP1 antibodies were used and gave similar results. The two DNA replication experimets shown in the supplementary information (Suppl Fig 13a/b) used two replicates (both individually depicted) that showed similar results with two independent anti-TopBP1 antibodies. Xenopus egg extracts were pre-tested before complementation experiments for replication capacity. Using tested extracts all attenpts to replicate results were successful. APEX2-proximity biotinylations and chromass experiments were done from four biological replicates and analysed by extensive statistical testing as inicated in the methods section. We show 3 such experiments that showed a high degree of overlap. Relevant hits that were not found in all three replicates (probably due to low abundance) are discussed in the manuscript. Biochemical binding experiments were replicated at least once. Our CHROMASS experiment (Fig 6) was done on the basis of 4 independent replicate samples that showed a high degree of overlap. Statistics(2-sided Student's test) was used to extract statistically significant hits.
Randomization	The techniques used do not benefit from randomisation

Blinding | The types of techniques used do not require blinding, because they don't dependent on researcher's interpretation.

Reporting for specific materials, systems and methods

We require information from authors about some types of materials, experimental systems and methods used in many studies. Here, indicate whether each material, system or method listed is relevant to your study. If you are not sure if a list item applies to your research, read the appropriate section before selecting a response.

Materials & experimental systems

n/a	Involved in the study
☐	☒ Antibodies
☐	☒ Eukaryotic cell lines
☐	☐ Palaeontology and archaeology
☐	☒ Animals and other organisms
☐	☐ Clinical data
☐	☐ Dual use research of concern
☐	☐ Plants

Methods

n/a	Involved in the study
☒	☐ ChIP-seq
☐	☐ Flow cytometry
☐	☐ MRI-based neuroimaging

Antibodies

Antibodies used

Anti-DB4, human TopBP1-1-360, rabbit, self-made
Anti- human Sld5-1-100, rabbit monoclonal ERP9318, Abcam ab139683
Anti-Flag peptide, mouse, clone M2, Sigma F1804
Anti-human 53BP1-350-400, rabbit, Invitrogen PA1-16565
Anti-Myc tag, mouse, clone 9E10
Anti- Xenopus TopBP1-1-365 (no. 1), rabbit, self-made
Anti- XTopBP1-999-1299 (no. 2), rabbit, self-made,
Anti- Xenopus Cdc45/ rabbit, self-made
Anti-Xenopus Sld5, rabbit, self-made
Anti-Xenopus PolE2, rabbit, self-made
Anti-Mcm6-775-821, rabbit, BETHYL A300-194A
Anti-Mcm4 -750-850, rabbit, Abcam ab4459
Anti-MBP tag, mouse, New England BioLabs E8032S

Validation

Anti-DB4: RNAi and co-IP with Treslin/TICRR and western blotting
anti human Sld5: rec protein and western blotting
Flag-peptide: Flag peptide IP and western blotting
53BP1: co-IP with TopBP1 and western blotting
Anti-Myc tag: IP of Myc-TopBP1 and western blotting
Anti-Xenopus TopBP1- no1 and 2: IP and cross antibody western blotting; immunodepletion from Xenopus extracts and rescue by rec TopBP1
Anti-XCdc45 and anti-XSld5: geminin-sensitive chromatin association from Xenopus egg extracts
Anti-Mcm4 and 6: geminin-sensitive loading on chromatin in Xenopus egg extracts

Eukaryotic cell lines

Policy information about cell lines and Sex and Gender in Research

Cell line source(s) | HEK293T cells ATCC CRL-11268; SF9 cells CRL-1711

Authentication | Cells showed typical microscopic appearance; because cells were not used for physiological experiments no detailed molecular authentication was required

Mycoplasma contamination | negative in regular mycoplasma testing using routine commercial tests

Commonly misidentified lines (See ICLAC register) | not applicable

Palaeontology and Archaeology

Specimen provenance | *Provide provenance information for specimens and describe permits that were obtained for the work (including the name of the issuing authority, the date of issue, and any identifying information). Permits should encompass collection and, where applicable, export.*

Specimen deposition | *Indicate where the specimens have been deposited to permit free access by other researchers.*

Dating methods | *If new dates are provided, describe how they were obtained (e.g. collection, storage, sample pretreatment and measurement), where they were obtained (i.e. lab name), the calibration program and the protocol for quality assurance OR state that no new dates are provided.*

☐ Tick this box to confirm that the raw and calibrated dates are available in the paper or in Supplementary Information.

Ethics oversight | *Identify the organization(s) that approved or provided guidance on the study protocol, OR state that no ethical approval or guidance was required and explain why not.*

Note that full information on the approval of the study protocol must also be provided in the manuscript.

Animals and other research organisms

Policy information about studies involving animals; ARRIVE guidelines recommended for reporting animal research, and Sex and Gender in Research

Laboratory animals | *For laboratory animals, report species, strain and age OR state that the study did not involve laboratory animals.*

Wild animals | *Provide details on animals observed in or captured in the field; report species and age where possible. Describe how animals were caught and transported and what happened to captive animals after the study (if killed, explain why and describe method; if released, say where and when) OR state that the study did not involve wild animals.*

Reporting on sex | *Indicate if findings apply to only one sex; describe whether sex was considered in study design, methods used for assigning sex. Provide data disaggregated for sex where this information has been collected in the source data as appropriate; provide overall numbers in this Reporting Summary. Please state if this information has not been collected. Report sex-based analyses where performed, justify reasons for lack of sex-based analysis.*

Field-collected samples | *For laboratory work with field-collected samples, describe all relevant parameters such as housing, maintenance, temperature, photoperiod and end-of-experiment protocol OR state that the study did not involve samples collected from the field.*

Ethics oversight | *Identify the organization(s) that approved or provided guidance on the study protocol, OR state that no ethical approval or guidance was required and explain why not.*

Note that full information on the approval of the study protocol must also be provided in the manuscript.

Clinical data

Policy information about clinical studies

All manuscripts should comply with the ICMJE guidelines for publication of clinical research and a completed CONSORT checklist must be included with all submissions.

Clinical trial registration | *Provide the trial registration number from ClinicalTrials.gov or an equivalent agency.*

Study protocol | *Note where the full trial protocol can be accessed OR if not available, explain why.*

Data collection | *Describe the settings and locales of data collection, noting the time periods of recruitment and data collection.*

Outcomes | *Describe how you pre-defined primary and secondary outcome measures and how you assessed these measures.*

Dual use research of concern

Policy information about dual use research of concern

Hazards

Could the accidental, deliberate or reckless misuse of agents or technologies generated in the work, or the application of information presented in the manuscript, pose a threat to:

No	Yes	
☒	☐	Public health
☒	☐	National security
☒	☐	Crops and/or livestock
☒	☐	Ecosystems
☒	☐	Any other significant area

Experiments of concern

Does the work involve any of these experiments of concern:

No	Yes	
☒	☐	Demonstrate how to render a vaccine ineffective
☒	☐	Confer resistance to therapeutically useful antibiotics or antiviral agents
☒	☐	Enhance the virulence of a pathogen or render a nonpathogen virulent
☒	☐	Increase transmissibility of a pathogen
☒	☐	Alter the host range of a pathogen
☒	☐	Enable evasion of diagnostic/detection modalities
☒	☐	Enable the weaponization of a biological agent or toxin
☒	☐	Any other potentially harmful combination of experiments and agents

Flow Cytometry

Plots

Confirm that:

☐ The axis labels state the marker and fluorochrome used (e.g. CD4-FITC).

☐ The axis scales are clearly visible. Include numbers along axes only for bottom left plot of group (a 'group' is an analysis of identical markers).

☐ All plots are contour plots with outliers or pseudocolor plots.

☐ A numerical value for number of cells or percentage (with statistics) is provided.

Methodology

Sample preparation: not applicable for this study

Instrument: not applicable for this study

Software: not applicable for this study

Cell population abundance: not applicable for this study

Gating strategy: not applicable for this study

☐ Tick this box to confirm that a figure exemplifying the gating strategy is provided in the Supplementary Information.

Magnetic resonance imaging

Experimental design

Design type: not applicable for this study

Design specifications: not applicable for this study

Behavioral performance measures: not applicable for this study

Acquisition

Imaging type(s): not applicable for this study

Field strength: not applicable for this study

Sequence & imaging parameters: not applicable for this study

Area of acquisition: not applicable for this study

Diffusion MRI ☐ Used ☐ Not used

Preprocessing

Preprocessing software	not applicable for this study
Normalization	not applicable for this study
Normalization template	not applicable for this study
Noise and artifact removal	not applicable for this study
Volume censoring	not applicable for this study

Statistical modeling & inference

Model type and settings	not applicable for this study
Effect(s) tested	not applicable for this study

Specify type of analysis: ☐ Whole brain ☐ ROI-based ☐ Both

Statistic type for inference (See Eklund et al. 2016)	not applicable for this study
Correction	not applicable for this study

Models & analysis

n/a	Involved in the study
☒	☐ Functional and/or effective connectivity
☒	☐ Graph analysis
☒	☐ Multivariate modeling or predictive analysis
